# Ecomorphology of the pectoral girdle in anurans (Amphibia, Anura): Shape diversity and biomechanical considerations

**DOI:** 10.1002/ece3.6784

**Published:** 2020-09-17

**Authors:** Karolin Engelkes, Lena Kath, Thomas Kleinteich, Jörg U. Hammel, André Beerlink, Alexander Haas

**Affiliations:** ^1^ Center of Natural History (CeNak) Universität Hamburg Hamburg Germany; ^2^ TPW Prüfzentrum GmbH Neuss Germany; ^3^ Institute of Materials Research Helmholtz‐Zentrum Geesthacht Geesthacht Germany; ^4^ Institut für Zoologie und Evolutionsforschung mit Phyletischem Museum, Ernst‐Hackel‐Haus und Biologiedidaktik Friedrich‐Schiller‐Universität Jena Jena Germany; ^5^ YXLON International GmbH Hamburg Germany

**Keywords:** locomotion, many‐to‐one mapping, muscle moment arm, stress dissipation, trade‐off

## Abstract

Frogs and toads (Lissamphibia: Anura) show a diversity of locomotor modes that allow them to inhabit a wide range of habitats. The different locomotor modes are likely to be linked to anatomical specializations of the skeleton within the typical frog Bauplan. While such anatomical adaptations of the hind limbs and the pelvic girdle are comparably well understood, the pectoral girdle received much less attention in the past. We tested for locomotor‐mode‐related shape differences in the pectoral girdle bones of 64 anuran species by means of micro‐computed‐tomography‐based geometric morphometrics. The pectoral girdles of selected species were analyzed with regard to the effects of shape differences on muscle moment arms across the shoulder joint and stress dissipation within the coracoid. Phylogenetic relationships, size, and locomotor behavior have an effect on the shape of the pectoral girdle in anurans, but there are differences in the relative impact of these factors between the bones of this skeletal unit. Remarkable shape diversity has been observed within locomotor groups indicating many‐to‐one mapping of form onto function. Significant shape differences have mainly been related to the overall pectoral girdle geometry and the shape of the coracoid. Most prominent shape differences have been found between burrowing and nonburrowing species with headfirst and backward burrowing species significantly differing from one another and from the other locomotor groups. The pectoral girdle shapes of burrowing species have generally larger moment arms for (simulated) humerus retractor muscles across the shoulder joint, which might be an adaptation to the burrowing behavior. The mechanisms of how the moment arms were enlarged differed between species and were associated with differences in the reaction of the coracoid to simulated loading by physiologically relevant forces.

## Graphical abstract

The relationships between locomotor behavior, shape, and function of the anuran pectoral girdle were investigated. Adaptation to burrowing behavior was observed and could be explained by biomechanical requirements. Other locomotor groups showed minor or no shape differences; large shape diversity within those groups indicated many‐to‐one mapping of form onto function.

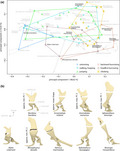

## 1 INTRODUCTION

Frogs and toads (Lissamphibia: Anura) inhabit a wide range of habitats and, among other things, utilize different locomotor behaviors within those habitats (Wells, 2007). Almost all anurans are capable of some kind of hopping or jumping (Wells, 2007) and swimming (Abourachid & Green, 1999). Some species have been reported to extensively use quadrupedal walking (e.g., Ahn, Furrow, & Biewener, 2004); other, in particular, fossorial species show burrowing behavior by moving the substrate using either their hind legs, forelegs, or heads (e.g., Emerson, 1976; Nomura, Rossa‐Feres, & Langeani, 2009). Arboreal species are able to climb in vegetation (Herrel et al., 2013); some of them have evolved parachuting or gliding abilities (Oliver, 1951).

From an ecomorphological point of view, different behaviors and the associated performances provide the link between the morphology of a specimen and its ecology (e.g., Arnold, 1983; Ricklefs & Miles, 1994; Wainwright, 1994). The anatomy determines the functional properties, which in turn determine the performance capacities of a specimen (Wainwright, 2007). In this context, natural selection should favor anatomical peculiarities that allow high locomotor performances, as moving in space is crucial for individuals, for example, to use the resources of their habitat, to encounter mates, or to escape from predators (e.g., Liedvogel, Chapman, Muheim, & Åkesson, 2013; Nathan et al., 2008).

Previous studies (e.g., Citadini, Brandt, Williams, & Gomes, 2018; Emerson, 1988; Vera, Ferretti, Abdala, & Cointry, 2020; Zug, 1978) have reported associations of anatomical traits with locomotor behavior or performance, or ecology within the Anura. Most of these studies have focused on the pelvic girdle, the relative length of fore‐ or hind limbs, or the anatomy of the hind limbs. For example, the takeoff speed for jumping was found to be associated with hind limb length, hind limb muscle mass, and muscle contraction rates (Choi & Park, 1996; Choi, Shim, & Ricklefs, 2003) and specific locomotor modes tended to be associated with differences in the shape of the sacrum (Jorgensen & Reilly, 2013). The mechanical properties of the femur and tibiofibula differed between certain locomotor modes (Vera et al., 2020; Wilson, Espinoza, Shah, & Blob, 2009). Species inhabiting the same microhabitat were similar with regard to their hind limb morphology, external body proportions, and performance in selected ecologically relevant tasks (Moen, Irschick, & Wiens, 2013).

High jumping performance, for instance, was generally associated with relatively short forelimbs (Zug, 1972), comparably long hind limbs (e.g., Astley, 2016; Emerson, 1978) with tibiofibulae being longer than the femora (Gómez & Lires, 2019), larger hind limb muscles (e.g., Astley, 2016), and specific physiological muscle properties (e.g., Astley, 2016; Chadwell, Hartwell, & Peters, 2002). The difference in the length of the hind limbs compared to the forelimbs was less pronounced in primary walking species (Reynaga, Astley, & Azizi, 2018). Jumping and walking, hopping species have been reported to differ in the anatomy of the ilio‐sacral joint and the associated configuration of the ilio‐lumbaris muscle, although there were some exceptions in the correlation of joint anatomy with locomotor mode (Emerson, 1979).

In addition, previously recognized morphological adaptations to swimming involved specific relative limb proportions (Gómez & Lires, 2019) and extensive foot webbing (Laurent, 1964). Additionally, the relative muscle mass of the hind limbs in frequently swimming species was higher if compared to other species (Moen, 2019). The ilio‐sacral joint in the aquatic species *Xenupus laevis* allowed for sliding and was thought to thereby increase the length of the power stroke and to contribute to fast submerging after breathing (Videler & Jorna, 1985).

Climbing behavior was usually associated with a bicondylar sacro‐urostylic articulation (Reilly & Jorgensen, 2011), large finger and toe tips (Moen et al., 2013), adhesive toe pads (Emerson & Diehl, 1980; Noble & Jaeckle, 1928), and modifications of the finger extensor muscles (Burton, 1998). In addition, hands and feet could be webbed (Laurent, 1964), the distal forelimbs of certain species might be adapted to grasping (Manzano, Abdala, & Herrel, 2008), and the presence of an intercalary cartilage or bone between the two terminal phalanges in some arboreal anuran species was thought to increase the efficiency of the adhesive toe pads (Noble & Jaeckle, 1928).

Finally, the body of burrowing species was generally observed to be globular (Dutta & Pradhan, 1985; Laurent, 1964) with relatively shorter and stronger limbs (Laurent, 1964; Moen, 2019) and a short tibiofibula relative to the femur (Enriquez‐Urzelai, Montori, Llorente, & Kaliontzopoulou, 2015; Gómez & Lires, 2019). Most backward burrowing species had enlarged metatarsal tubercles (Kley & Kearney, 2006; Moen et al., 2013). Short hind limbs and the presence of metatarsal tubercles have been suggested to increase the performance of backward burrowing (Emerson, 1976). Further examples of the adaptation to backward burrowing include the increase in the size and robustness of the prehallux (Kley & Kearney, 2006) and species‐specific modification of the feet muscles (Blotto, Pereyra, Faivovich, Dias, & Grant, 2017; Burton, 2001; Sanders & Davies, 1983). Headfirst burrowing has been reported to be species‐specifically associated with a modified skull (Davies, 1984; Menzies & Tyler, 1977), massive mandibles (Menzies & Tyler, 1977), relatively short and robust forelimbs (Brown, Jackson, & Brown, 1972), or modifications of the manus (Kley & Kearney, 2006).

The forelimbs of anurans have been reported to accomplish species‐ and case‐specific tasks during locomotion (e.g., hopping/jumping: Nauwelaerts & Aerts, 2006; swimming: Abourachid & Green, 1999; Gillis & Biewener, 2000; walking: Reynaga et al., 2018; burrowing: Sanders & Davies, 1983; climbing: Manzano et al., 2008). The forelimbs, for example, decelerate the body during coordinated landing (Cox & Gillis, 2015), move the soil during headfirst burrowing (Emerson, 1976), or stabilize the body during gliding (Emerson & Koehl, 1990). In addition, some muscles originating from the pectoral girdle and inserting onto the forelimb have been shown to be active during different phases of a jump (Akella & Gillis, 2011). Yet, the pectoral girdle, that is, the central element linking the forelimbs to the axial skeleton, has received little attention regarding the association of anatomical traits with and the functional adaptation to different locomotor behaviors.

Different pectoral girdle types (arciferal, firmisternal) were suggested to accomplish similar tasks (i.e., dissipating landing forces), but in different ways (Emerson, 1983, 1984). One previous study reported that higher jumping abilities were associated with shorter scapulae (Zug, 1972), whereas another observed jumping species to have long scapulae with broad proximal and distal ends, and long claviculae and coracoids (Soliz, Tulli, & Abdala, 2017). Headfirst burrowing was associated with a forward shifted scapula causing the suprascapula to overlap the posterior margin of the skull, and robust and posteromedially directed coracoids in some species (Davies, 1984; Emerson, 1976). Besides these partly contradictory reports, little is known about the anuran pectoral girdle in relation to different locomotor behaviors and on the biomechanical functions of this skeletal complex during locomotion.

Here, we aim to resolve the relationships between locomotor mode, shape variation, and biomechanical function of the pectoral girdle of anurans. To do so, selected anuran species were assigned to one of six groups of locomotor behavior (subsequently called locomotor groups) and the shape of their pectoral girdle bones was assessed by means of geometric morphometrics. The phylogenetic signal was determined, and shape differences among locomotor groups were statistically assessed. The pectoral girdles of selected species were analyzed with regard to the effects of shape differences on muscle moment arms across the shoulder joint and simulated stress dissipation within the coracoid. Results were discussed in the context of adaptation to locomotor behaviors.

## 2 MATERIAL AND METHODS

### 2.1 Specimens and µCT scanning

**FIGURE 1:**
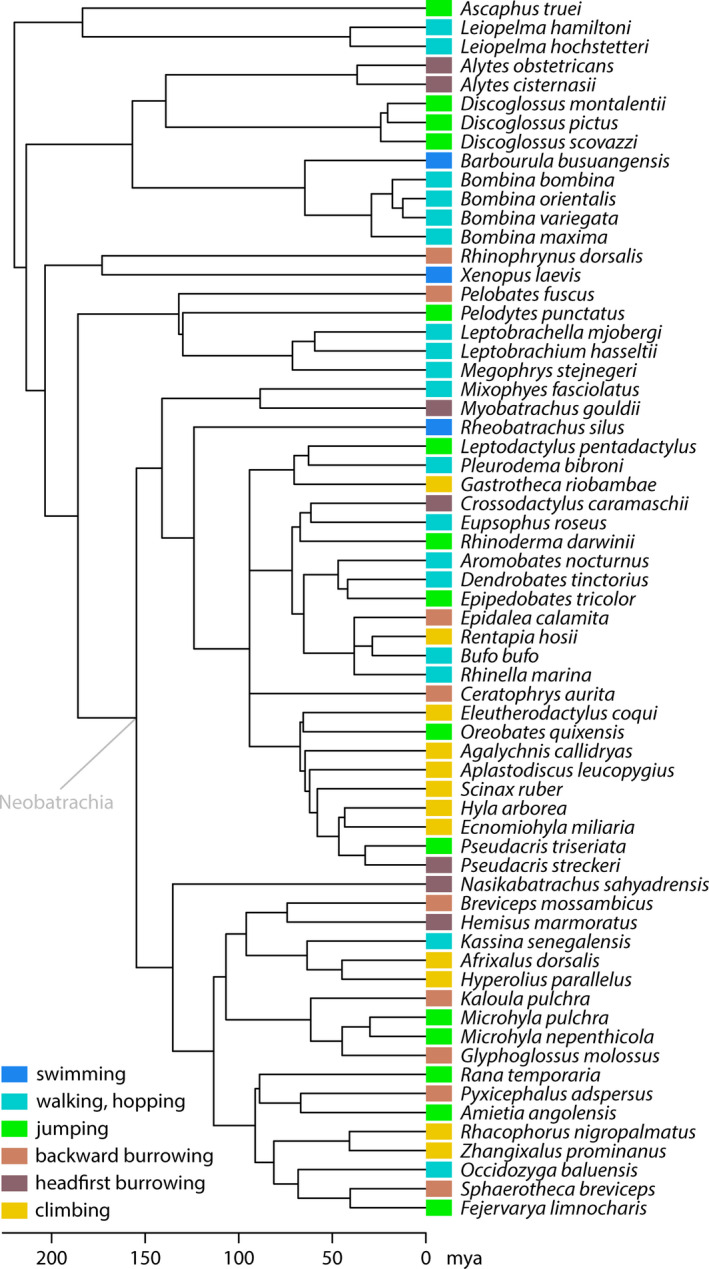
Phylogenetic relation and locomotor behavior of species examined in this study

Locomotor groups were defined (Table 1) and assigned based on literature accounts (Appendix S1: Tables A1, A2). Sixty‐four species (Figure 1) covering 31 of the 52 currently recognized (Frost, 2020) anuran (Amphibia: Anura) families were selected based on their phylogenetic position and locomotor behavior. A time‐calibrated phylogeny was extracted from TimeTree.org (accessed 2nd March 2020; Kumar, Stecher, Suleski, & Hedges, 2017); six species were replaced by close relatives (assessed from Pyron & Wiens, 2011) for extraction as they were not listed on TimeTree.org. Species names were updated following Frost (2020). The aim was to achieve heterogeneous subclades with regard to locomotor behavior and a wide dispersion of locomotor groups across the phylogeny in order to avoid potential negative effects on the statistical analyses (Adams & Collyer, 2018).

**TABLE 1:** Definition of locomotor groups

Locomotor group	Definition
Swimming	Purely aquatic locomotion.
Walking, hopping	Quadrupedal walking or hopping (sensu Emerson, 1979: jumps with a maximum length of less than 8–9 times snout–vent length) on land. Optional swimming behavior, no climbing or burrowing.
Jumping	Same as “walking, hopping” but with maximum jumps longer than 8–9 times snout–vent length (Emerson, 1979).
Backward burrowing	Swimming, walking, hopping, or jumping but with additional digging using the hind limbs. No use of arms/head for digging.
Headfirst burrowing	Same as “backward burrowing” but additional use of forelimbs or head to move soil.
Climbing	Swimming, walking, hopping, or jumping but with additional climbing and jumping locomotion in vegetation. Optional parachuting or gliding locomotion (sensu Oliver, 1951: while falling descending along path that deviates less [parachuting] or more [gliding] than 45° from the vertical).

Selected micro‐computed tomography (µCT) scans of a previous study (Engelkes et al., 2019) were used in combination with additional µCT volumes. Scans were performed with a Skyscan 1172 (Bruker microCT), Phoenix Nanotom S or M (GE Sensing & Inspection Technologies GmbH), Phoenix v|tome|x L 450 (GE Sensing & Inspection Technologies GmbH), or a YXLON FF20 CT or FF35 CT (YXLON International GmbH; Appendix S1: Table A1). Additional µCT volumes were downloaded from MorphoSource (https://www.morphosource.org/; Appendix S1: Table A2).

### 2.2 Segmentation and surface generation

A previous study (Engelkes et al., 2019) found that the techniques applied to generate the polygon surfaces have a significant effect on the landmark data acquired from them. The workflow herein followed the recommendations in Engelkes (in review) in order to obtain surfaces that are as accurate as possible. The pectoral girdle bones (including calcified sternal or episternal elements, if applicable) were roughly segmented in Amira (version 6.0.1; Konrad‐Zuse‐Zentrum Berlin, FEI Visualization Sciences Group), and the mean gray value *m* of pectoral girdle bones and surrounding soft tissues, and the standard deviation of the soft tissue gray values were determined for each original CT volume separately. The mean gray value *m* was used to set limits to the gray value histogram of the respective CT volume in Fiji (based on ImageJ 1.51n; Schindelin et al., 2012; Schneider, Rasband, & Eliceiri, 2012). The limits were chosen such that they laid symmetrically around the value calculated by 1.019 × *m* − 462.812 (see Engelkes, in review for the derivation of this formula) and such that the contrast of bone and surrounding voxels was maximized without bone voxels getting black.

Each adjusted CT volume was resliced from top to bottom and from left to right, and all stacks were thresholded by automatic local thresholding (Fiji plugin *Auto Local Threshold*, Landini, Rueden, Schindelin, Hiner, & Helfrich, https://imagej.net/Auto_Local_Threshold). The three thresholding results of each CT volume were combined in Amira by setting those voxels as bone that were classified as bone in any two of the three thresholded stacks. The resulting stack was combined with the rough segmentation of the pectoral girdle bones to separate the bones form other structures. Foramina were filled and artifacts (i.e., segmented noise, unsegmented bone voxels) were corrected in regions in which semilandmarks should be placed by manually adjusting the segmentation accordingly.

Polygon surfaces were generated using the *Generate Lego Surface* module in Amira in combination with surface simplification (reduction of polygon count and smoothing; *Simplification Editor* and *Smooth Surface* module) to a subjective optimal degree. Surface generation and simplification were accelerated by a modified version of the *MultiExport* macro (Engelkes, Friedrich, Hammel, & Haas, 2018). The bones of the right pectoral girdle halves were mirrored (MeshLab version 1.3.3; Cignoni et al., 2008) to the left to avoid any potential bias due to orientation during landmark acquisition. Surfaces with major deformations or artifacts were excluded from subsequent steps.

### 2.3 Landmarks and superimposition

**FIGURE 2:**
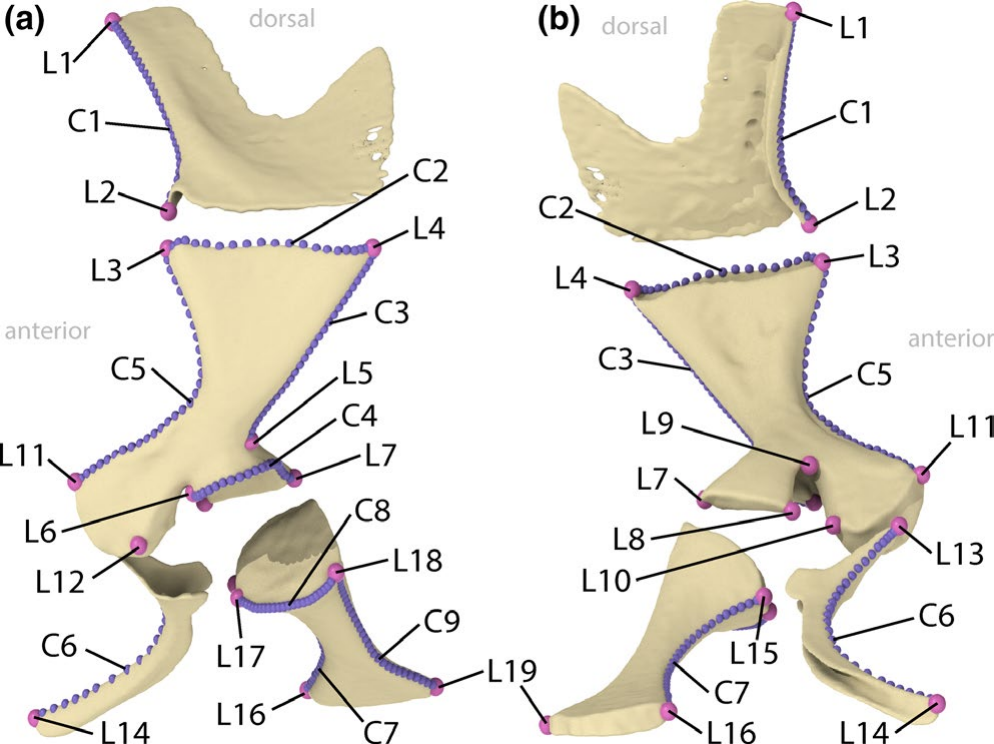
Landmarks (pink; L1‐19) and semilandmarks (violet; C1‐9) on the bones of the left‐side pectoral girdle of *Ecnomiohyla miliaria*. (a) Lateral view. (b) Medial view

Landmarks were, with slight modifications, adopted from Engelkes et al. (2019) and complemented by curves of sliding semilandmarks (Gunz & Mitteroecker, 2013; Figure 2; Appendix S1: Table A3). For each pectoral girdle half, 19 fixed landmarks (including start and end points of curves) and nine curves with 21 to 29 semilandmarks were acquired in Stratovan Checkpoint (version 2020.02.05.1043; Stratovan Corporation). No landmarks were acquired from the sternum or episternum, as those structures were present in only some species. Three microhylid species (*Kaloula pulchra*, *Microhyla nepenthicola*, and *M. pulchra*) lacked a clavicula and, consequently, the (semi)landmarks on the clavicula were missing in the respective landmark configurations.

All subsequent steps were performed in R (version 3.5.3; R Core Team, 2019) using RStudio (version 1.1.463; RStudio Team, 2018) and functions of the packages abind (version 1.4‐5; Plate & Heiberger, 2016), ape (version 5.3; Paradis & Schliep, 2018), geomorph (version 3.2.1; Adams, Collyer, & Kaliontzopoulou, 2020), Morpho (version 2.7; Schlager, 2017), rgl (version 0.100.47; Adler & Murdoch, 2020), RRPP (version 0.5.2; Collyer & Adams, 2018, 2020), shapes (version 1.2.5; Dryden, 2019), and vegan (version 2.5‐4; Oksanen et al., 2019). The landmark sets were imported into R, and the missing (semi)landmarks were estimated (*estimate.missing*) to allow for the incomplete landmark sets being analyzed together with the others. The following five subsets of (semi)landmarks were defined: all fixed landmarks (including start and endpoints of curves) to analyze the overall geometry of the pectoral girdle, and all landmarks and semilandmarks of a given pectoral girdle bone to allow for a more detailed shape comparison. Species lacking a clavicula were excluded from the subset consisting of (semi)landmarks on the clavicula. The following steps were performed for the full landmark sets and for each subset separately. All landmark sets of a given species were superimposed using a Generalized Procrustes Analysis (GPA; *gpagen,* if applicable, including sliding of semilandmarks to minimize bending energy), rescaled to their original centroid size and the species mean shape was calculated (*mshape*). A GPA (including sliding of semilandmarks to minimize bending energy, if applicable) was performed to superimpose the species mean shapes. The resulting sets of superimposed species mean shapes will subsequently be referred to as full landmark dataset and landmark datasets i through v, with the full dataset consisting of all landmarks and semilandmarks, landmark datasets i denoting the set of fixed landmarks and ii‐v denoting the sets comprising all landmarks and semilandmarks, respectively, on the scapula, coracoid, cleithrum, or clavicula.

### 2.4 Statistical analyses and visualization

The full landmark dataset was used to assess the modularity (sensu Schlosser, 2002) within the pectoral girdle in a phylogenetic context by calculating the covariance ratio (*phylo.modularity*; Adams, 2016); modules are constituted by highly correlated subsets of traits (here landmark coordinates), whereas the covariation between such modules is relatively weak. The statistical significance was assessed by 1,000 permutations.

The following analyses were performed for each set of superimposed species mean shapes (landmark datasets i–v) separately. The phylogenetic signal in the landmark data was assessed using a multivariate version of the K‐statistic with the statistical significance being determined by 1,000 random permutations (*physignal*; Adams, 2014). As there was a statistically significant phylogenetic signal in all landmark datasets, separate phylogenetic MANOVAs (pMANOVAs; using residual randomization and type‐II sums of squares; *procD.pgls*) were performed to test for significant differences between the mean shapes of locomotor groups. Potential effects of specimen size on shape were accounted for by incorporating the log‐transformed centroid size and its interaction with mode of locomotion in the pMANOVAs. If there were statistically significant differences, pairwise comparisons of the mean shape between locomotor groups were performed while accounting for size (*pairwise*; null model: *coords ~ logCS*, where *coords* denotes one of landmark datasets i–v and *logCS* the log‐transformed centroid size). Statistical significance was assessed by 1,000 permutations in pMANOVAs and pairwise comparisons; *p*‐values below .05 were considered significant in all tests.

Principal component analyses (PCAs; *gm.prcomp*) were separately performed for landmark datasets i–v to visualize the distribution of species mean shapes in morphospace (*plot*, *shapeHulls*). For the dataset of fixed landmarks only (i), all individual landmark configurations belonging to a given species were transformed as their respective mean shape had been transformed during GPA and PCA (details in Engelkes et al., 2019); the transformed landmark configurations were plotted along with their means. The number of significant principal components was determined using the broken‐stick model (Macarthur, 1957; *evplot* function published with Borcard, Gillet, & Legendre, 2011). Surfaces and landmark configurations were rendered in MODO (version 10.1v2; The Foundry).

### 2.5 Muscle moment arms

**FIGURE 3:**
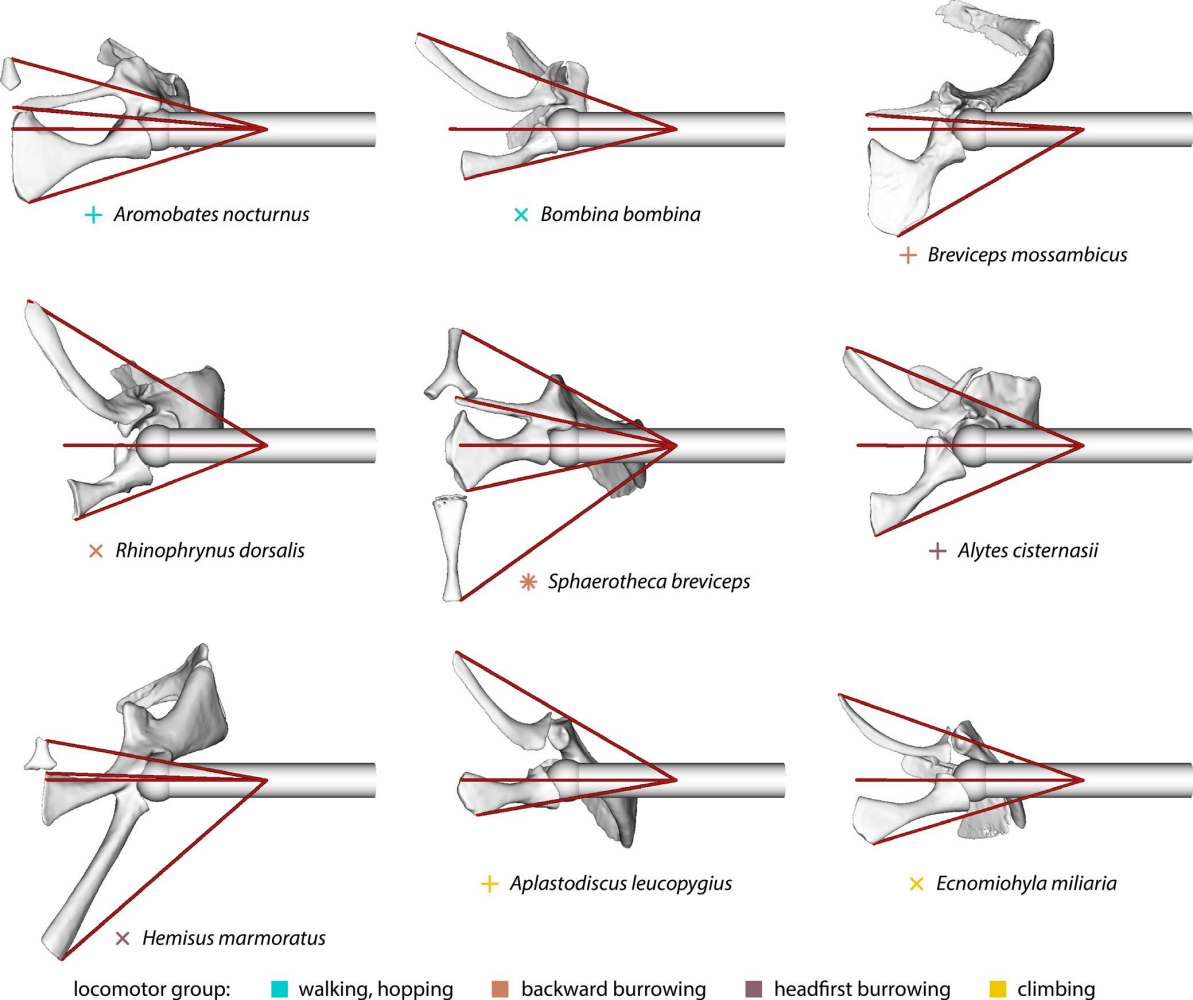
Musculoskeletal models of left‐side pectoral girdle bones of selected anuran specimens. Ventral views, anterior to the top, medial to the left. Warping objects not shown. Symbols and colors as in Figure 4

**FIGURE 4:**
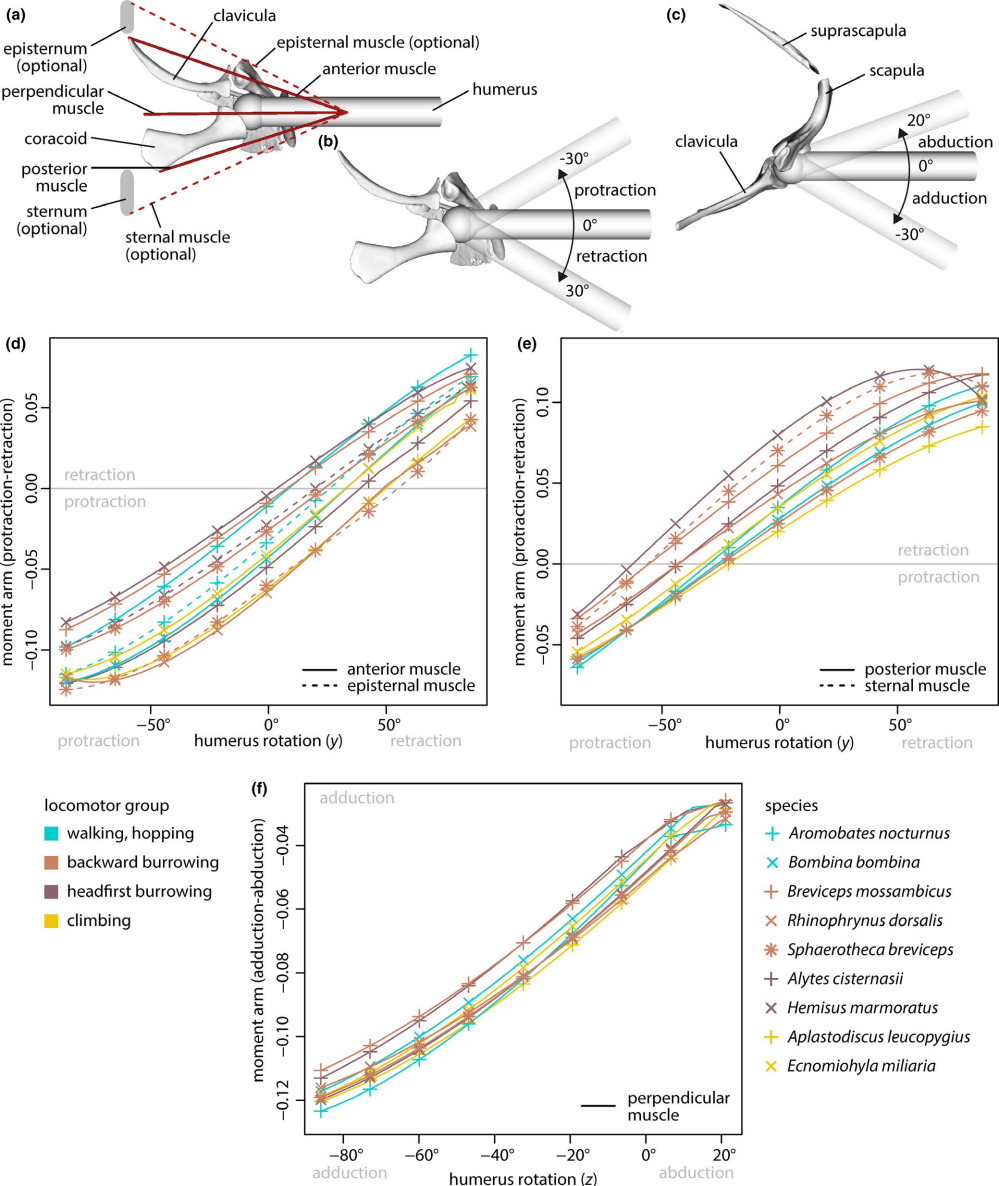
Hypothetical muscles analyzed in musculoskeletal models and respective muscle moment arms. Warping objects not shown. (a) Ventral view of musculoskeletal model of *Ecnomiohyla miliaria* with added structures that are optionally present in some specimens. (b) View of (a) without muscles to illustrate humerus protraction and retraction. (c) Anterior view of (b) to illustrate humerus adduction and abduction. (d) Moment arms of anterior and episternal muscles with regard to protraction and retraction. (e) Moment arms of posterior and sternal muscles with regard to protraction and retraction. (f) Moment arms of perpendicular muscle with regard to adduction and abduction

Musculoskeletal models were created for representative specimens of selected species (Figures 3 and 4) that appeared interesting based on their position in the morphospaces of the overall pectoral girdle shape (landmark dataset i) and of the coracoid shape (landmark dataset iii). The shape analyses suggested that most locomotor‐mode‐related shape differences occurred in the ventral part of the pectoral girdle. Therefore, the effects of the shape of the ventral pectoral girdle part (i.e., clavicula and coracoid) on the moment arms of muscles across the shoulder joint were assessed. Models were created in OpenSim (version 3.3; Delp et al., 2007) using simplified (inner structures removed, all holes in the surface closed, polygon count reduction and smoothing) surfaces of the respective specimens.

Both (left and right) landmark configurations of a given specimen were combined to one configuration. This configuration was used to transform the corresponding surfaces of the left‐side pectoral girdle bones and, if applicable, the bony part of the sternum or episternum to a common size and comparable orientation (R, MeshLab, MODO). The origin of the coordinate system was located in the shoulder joint cavity, the *y*‐*z*‐plane was parallel to the sagittal plane with the *z*‐axis being approximately parallel to the long axis of the specimen, and the line connecting the anteromedial tip of the clavicula to the posteromedial tip of the coracoid was parallel to the *x*‐*z*‐plane. All commonly scaled and orientated surfaces were equipped with the same simplified humerus in order to exclude any potential effects of the humerus shape on muscle moment arms. The shoulder joint was defined with two axes of rotation: one allowing adduction and abduction, and one allowing protraction and retraction. The humerus being aligned with the *x*‐axis (perpendicular to the sagittal [*y*‐*z*] plane) was used as reference position for angular measurements.

Previous studies (e.g., Bigalke, 1927; Gaupp, 1896; Ritland, 1955) showed that different muscles originated along the midline of the ventral side of the pectoral girdle and inserted onto the humerus. Those muscles were reduced to three hypothetical muscles that were included in each model (Figure 4a): one muscle (“anterior”) originating from the anteromedial tip of the clavicula, one (“perpendicular”) being perpendicular to the long axis of the specimen, and one (“posterior”) originating from the posteromedial tip of the coracoid. If an episternum or sternum was present and contained a pars ossea (senus Gaupp, 1896), additional muscles (“episternal,” “sternal,” respectively) originating from, respectively, the anterior or posterior tip of the bone were included, too. All muscles were defined to insert in a common point at the humerus. Warping objects were configured as needed to prevent muscle pathways from intersecting with skeletal elements; the potential effects of soft tissues in shaping muscle pathways were neglected. The moment arms of the perpendicular muscle was assessed with regard to adduction and abduction, the moment arms of all other muscles were determined with regard to protraction and retraction (Figure 4b, c).

### 2.6 Finite element analysis of coracoids

The species close to the extreme ends of the first two principal components and a species close to the mean shape in the landmark dataset of species mean coracoid shapes (iii) were chosen to assess the effects of different loading conditions by using finite element analyses. The coracoid surfaces were extracted from the corresponding surfaces used for musculoskeletal modeling. As a consequence of this approach, all coracoids were scaled and orientated in a way that reflected the actual conditions in the specimens and they were modeled as solid structures. Neglecting inner structures was expected to have a minor effect base on the observations of Mielke and Nyakatura (2019). The coracoid in *Hemisus marmoratus* was fused to other bones; those bones were manually removed (MODO).

**FIGURE 5:**
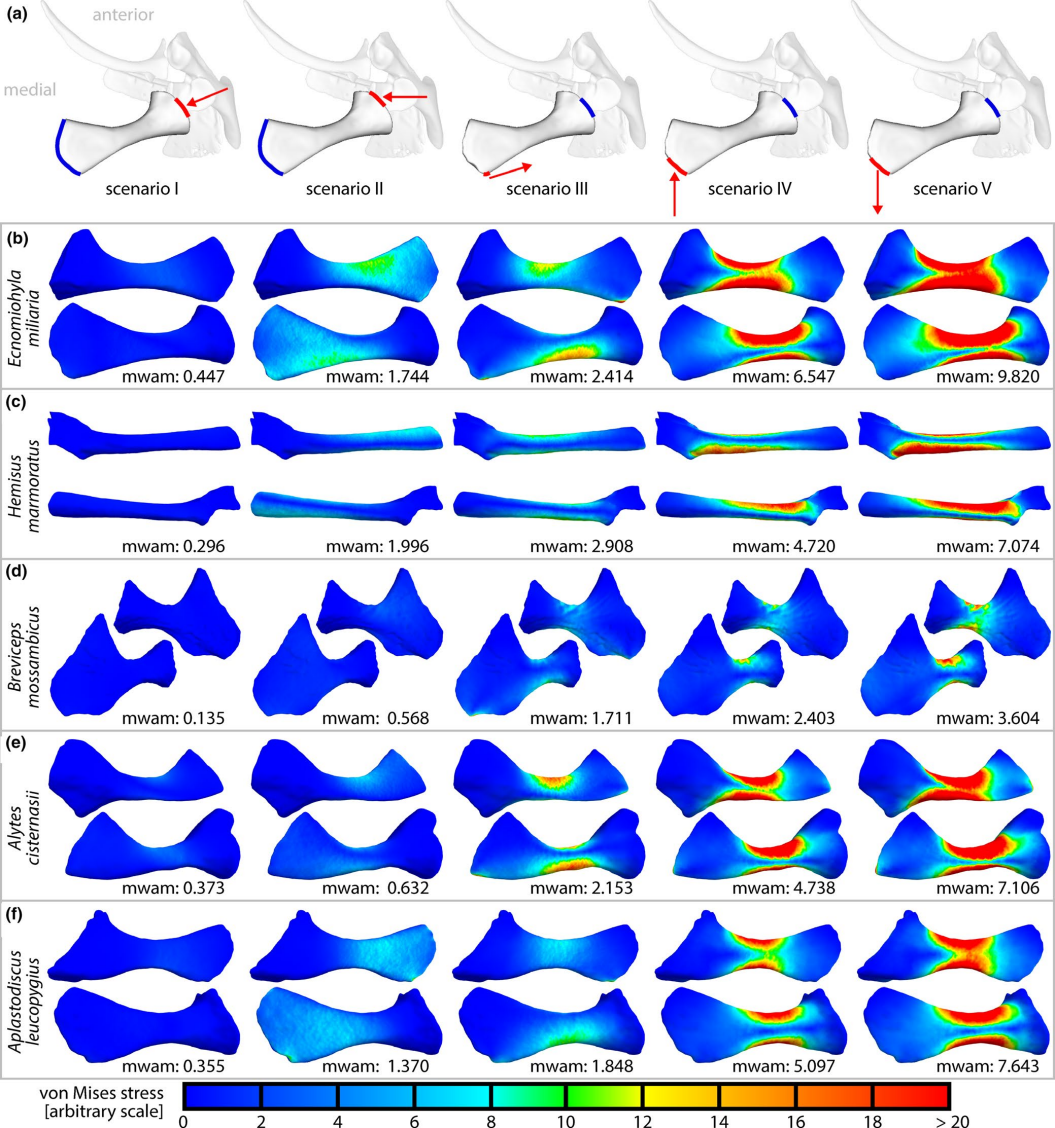
Loading scenarios and von Mises stresses in finite element analyses (FEAs) of coracoids. (a) Loading scenarios in ventral views. Blue line: surface fixed in space; red line: area of force application; red arrow: direction of applied force. (b–f) Results of FEAs. Size not comparable among species. Anterior approximately to the top; dorsal view above ventral view. mwam: mesh‐weighted arithmetic mean von Mises stress

Tetrahedral meshes were generated and the models were set up in FEBio Studio (version 1.0.0; Maas, Ellis, Ateshian, & Weiss, 2012). Bone was modeled as an isotropic elastic material with a Young's modulus of 10 GPa and a Poisson's ratio of 0.35 as these values lay within the previously reported ranges for vertebrate bones (e.g., Currey, 1984; Hudson, Bennett, & Franklin, 2004). Five different loading scenarios were deduced from supposed functions of the coracoid (Table 2; Figure 5a). The applied loads were scaled by the area on which they were applied such that equal forces were applied across all loading scenarios and specimens. Von Mises stresses were visualized in PostView (version 2.5.0; also part of FEBio suite), and the mesh‐weighted arithmetic mean von Mises stresses (mwam; Marcé‐Nogué, Esteban‐Trivigno, Escrig, & Gil, 2016) were calculated in R.

**TABLE 2:** Loading scenarios applied to selected coracoids

Scenario	Fixed in space	Force	Purpose
I	Medial surface (interface to epicoracoid cartilage)	Compressive load along the long axis (line connecting the mean point of the anteromedial and posteromedial tips of the coracoid with the center of rotation of the shoulder joint), applied to a part of the glenoidal surface	Reference condition, as we expected this to reflect the optimal loading direction
II	Medial surface	Compressive load, perpendicular to the sagittal plane, applied to a part of the glenoidal surface	Simulation of medially directed force components, that occur during landing (Emerson, 1983) or burrowing (Emerson, 1976)
III	Part of glenoidal surface	Load (tension) along the trajectory of the hypothetical posterior muscle (musculoskeletal model in reference position), applied to a small area on the posteromedial part of the coracoid	Simulation of loading due to muscles originating in this area
IV	Part of glenoidal surface	Anteriorly directed load, parallel to the longitudinal axis of the specimen, applied to the posteromedial part of the medial surface of the coracoid	Simulation of potential anteriorly directed force component that a sternum might transmit to the pectoral girdle if muscles attached to the sternum contract and thereby pull the sternum forward
V	Part of glenoidal surface	Posteriorly directed load, parallel to the longitudinal axis of the specimen, applied to the posteromedial part of the medial surface of the coracoid	Simulation of potential effect of a m. sterno‐epicoracoideus or m. rectus abdominis (Emerson, 1983; Jones, 1933) that could be attached to the posteromedial tip of the epicoracoid cartilage

## 3 RESULTS

### 3.1 Overall shape of pectoral girdle bones

The first five principal components (PCs) of the species mean shapes of the overall geometry of the pectoral girdle bones (landmark dataset i) were significant and, respectively, represented 48.62%, 14.42%, 8.5%, 5.62%, and 5.1% of the variance in the landmark data.

**FIGURE 6:**
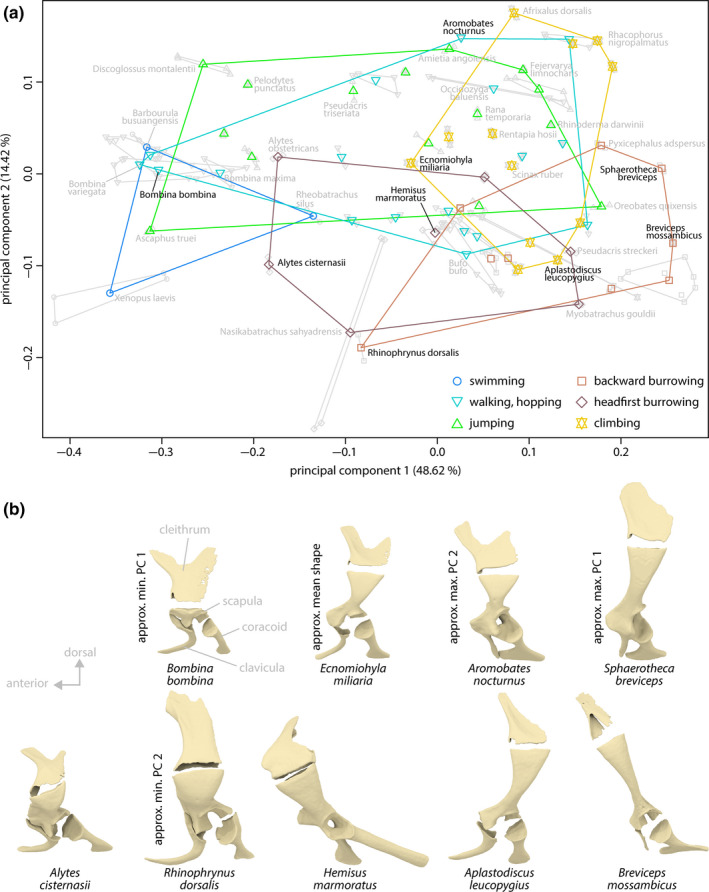
Principal component (PC) plot of overall species mean shapes of pectoral girdle bones (landmark dataset i) and surfaces of selected specimens. (a) PC plot of species mean shapes. Gray points illustrate single landmark configurations used to calculate the species mean shapes. (b) Surfaces of the left‐side pectoral girdles used for musculoskeletal modeling; sternal and episternal elements omitted

The pectoral girdle shapes of swimming and climbing species, and those of swimming and backward burrowing species differed with regard to the shape differences associated with PC 1 (Figure 6) and, in the latter case, also PC 4. In addition, there was a tendency for shape differences between backward and headfirst burrowing species along PCs 1 and 4, between burrowing and nonburrowing species along PC 2, and between backward burrowing and climbing species along PC 4. Yet, all locomotor groups comprised pectoral girdle shapes that were similar to some of those observed in other groups (i.e., all locomotor groups showed some regions of overlap along PCs 1–5 in pairwise comparisons) and the species represented by more than one specimen showed some shape similarities (overlap in PC plot) with other species.

The first principal component was mainly associated with differences in the height (dorsal–ventral expansion) of the scapula relative to its width (anterior–posterior expansion) and to the length of the clavicula and coracoid, the position of the glenoid cavity relative to the dorsal margin of the scapula and the ventral midline, as well as the angles of the clavicula and coracoid relative to each other and to the ventral midline. A high scapula was generally associated with a more medially located glenoid cavity and with comparably short clavicula and coracoid; the long axes of the ventral bones lay approximately parallel to each other and rather perpendicular to the ventral midline of the specimen. If a flat scapula was present, the ventral bones were angled such that their long axes diverged medially. The clavicula was curved, and the anterior and posterior margins of the coracoid were comparably straight (inferred from exemplary pectoral girdles shown in Figure 6b as semilandmarks were not included in this dataset). The second principal component was also associated with differences in the shape and angle of the ventral bones, the length of these bones relative to the height of the scapula, and the position (in anterior–posterior direction) of the glenoid cavity relative to the dorsal margin of the scapula.

The phylogenetic signal (*K*_mult_ = 0.9595; *p* = .001) and the effects of the log‐transformed centroid size and locomotor mode on shape were statistically significant (Table 3). The locomotor mode (*R* ^2^ = 0.19483) accounted for considerably more of the shape variation than the log‐transformed centroid size (*R* ^2^ = 0.05260). The pairwise comparison of mean shapes of locomotor groups revealed that climbing species significantly differed from swimming species, that the group of backward burrowing species significantly differed from headfirst burrowing species, and that each burrowing group significantly differed from all other locomotor groups, except for headfirst burrowers that did not differ from swimmers (Table 4).

**TABLE 3:** Results of pMANOVA of species mean shapes of pectoral girdle bones performed for fixed landmarks only (landmark dataset i)

	*Df*	SS	MS	*R* ^2^	*F*	*p*
Log. centroid size	1	0.0013047	0.00130473	0.05260	4.2706	.001*
Locomotor mode	5	0.0048325	0.00096649	0.19483	3.1635	.001*
Log. centroid size:locomotor mode	5	0.0017934	0.00035867	0.07230	1.1740	.234
Residuals	52	0.0158866	0.00030551	0.64052		
Total	63	0.0248028				

Note

Asterisks (*) denote statistical significance.

**TABLE 4:** Pairwise comparison of locomotor‐group‐specific species mean shapes of pectoral girdle bones (calculated from species mean shapes)

Locomotor groups compared	*p* value for overall shape	*p* value for scapula shape	*p* value for coracoid shape
Swimming–walking, hopping	.052	.091	.720
Swimming–jumping	.051	.216	.794
Swimming–backward burrowing	.006*	.023*	.265
Swimming–headfirst burrowing	.074	.024*	.918
Swimming–climbing	.018*	.012*	.828
Walking, hopping–jumping	.615	.321	.545
Walking, hopping–backward burrowing	.023*	.209	.006*
Walking, hopping–headfirst burrowing	.003*	.130	.058
Walking, hopping–climbing	.352	.055	.260
Jumping–backward burrowing	.002*	.025*	.001*
Jumping–headfirst burrowing	.001*	.027*	.179
Jumping–climbing	.116	.005*	.842
Backward burrowing–headfirst burrowing	.017*	.947	.001*
Backward burrowing–climbing	.014*	.975	.001*
Headfirst burrowing–climbing	.007*	.633	.194

Note

Asterisks (*) denote statistical significance.

The modularity test performed on the full landmark dataset revealed significant modularity (covariance ratio: 0.8133; *p* = .001).

### 3.2 Shape of the scapula

**FIGURE 7:**
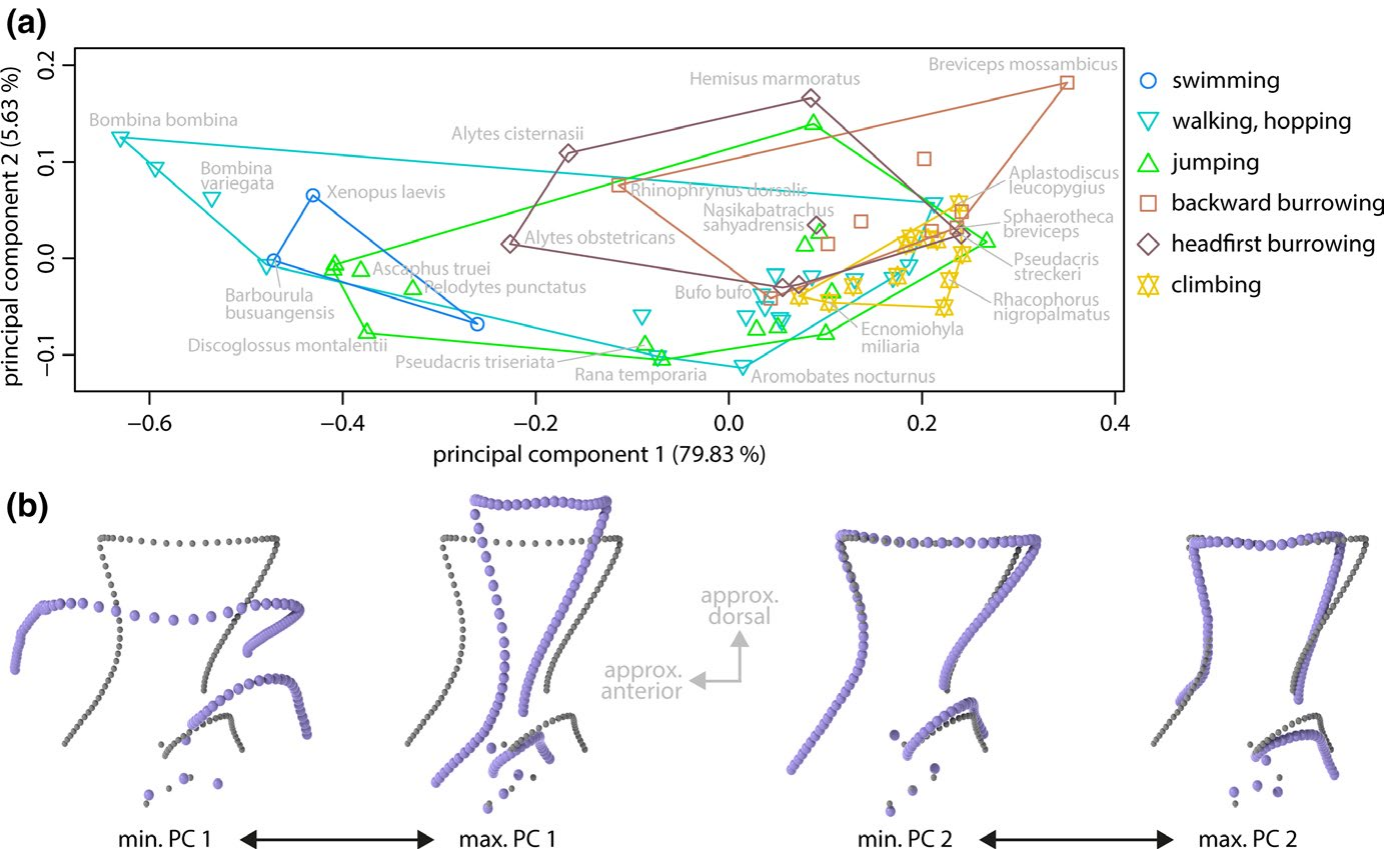
Principal component (PC) plot of species mean shapes of scapulae (landmark dataset ii) with extreme shapes along PCs. (a) PC plot of species mean shapes. (b) Extreme shapes of PC 1 in lateral view. (c) Extreme shapes of PC 2 in lateral view. Gray: mean shape; violet: extreme shape

Most shape variance (79.83%) in the species mean shapes of the scapula (landmark dataset ii) was represented by PC 1. This principal component was the only significant component and revealed a tendency toward shape differences between non‐neobatrachian and neobatrachian anurans (Figure 1; both groups roughly separated along PC 1 in Figure 7). It was associated with differences in the height relative to the width of the scapula, and with the curvature of the anterior margin. A high scapula was associated with a concavely shaped anterior margin, whereas the corresponding structure of a low scapula was rather convex. PC 2 represented 5.63% of the variance and, despite its insignificance, was mainly associated with differences in the torsion of the scapula around its long (dorsoventral) axis, the length of the dorsal margin relative to the ventral expansion, and the angle of the dorsal margin of the glenoid cavity relative to the horizontal plane. The scapula shape of swimming species differed from the shape of burrowing and climbing species along PC 1, and there was a tendency toward shape differences between burrowing and climbing species along PCs 1 and 2.

There was a strong and significant phylogenetic signal (*K*_mult_ = 1.6003; *p* = .001) in the species mean shapes of the scapula. The effects of the log‐transformed centroid size (*R* ^2^ = 0.04950) and the locomotor mode (*R* ^2^ = 0.17668) were statistically significant with the latter clearly exceeding the former (Table 5). The pairwise comparison of the mean shapes of locomotor groups (Table 4) revealed that jumping and swimming species significantly differed from burrowing and climbing species.

**TABLE 5:** Results of pMANOVA of species mean shapes of scapulae performed for respective fixed landmarks and semilandmarks (landmark dataset ii)

	*df*	SS	MS	*R* ^2^	*F*	*p*
Log. centroid size	1	0.0010460	0.00104602	0.04950	3.7193	.016*
Locomotor mode	5	0.0037336	0.00074672	0.17668	2.6551	.003*
Log. centroid size:locomotor mode	5	0.0009149	0.00018298	0.04329	0.6506	.872
Residuals	52	0.0146244	0.00028124	0.69203		
Total	63	0.0211326				

Note

Asterisks (*) denote statistical significance.

### 3.3 Shape of the coracoid

The first three PCs of the species mean shapes of the coracoid (landmark dataset iii) were significant and, respectively, represented 59.25%, 14.81%, and 9.15% of the total variance.

**FIGURE 8:**
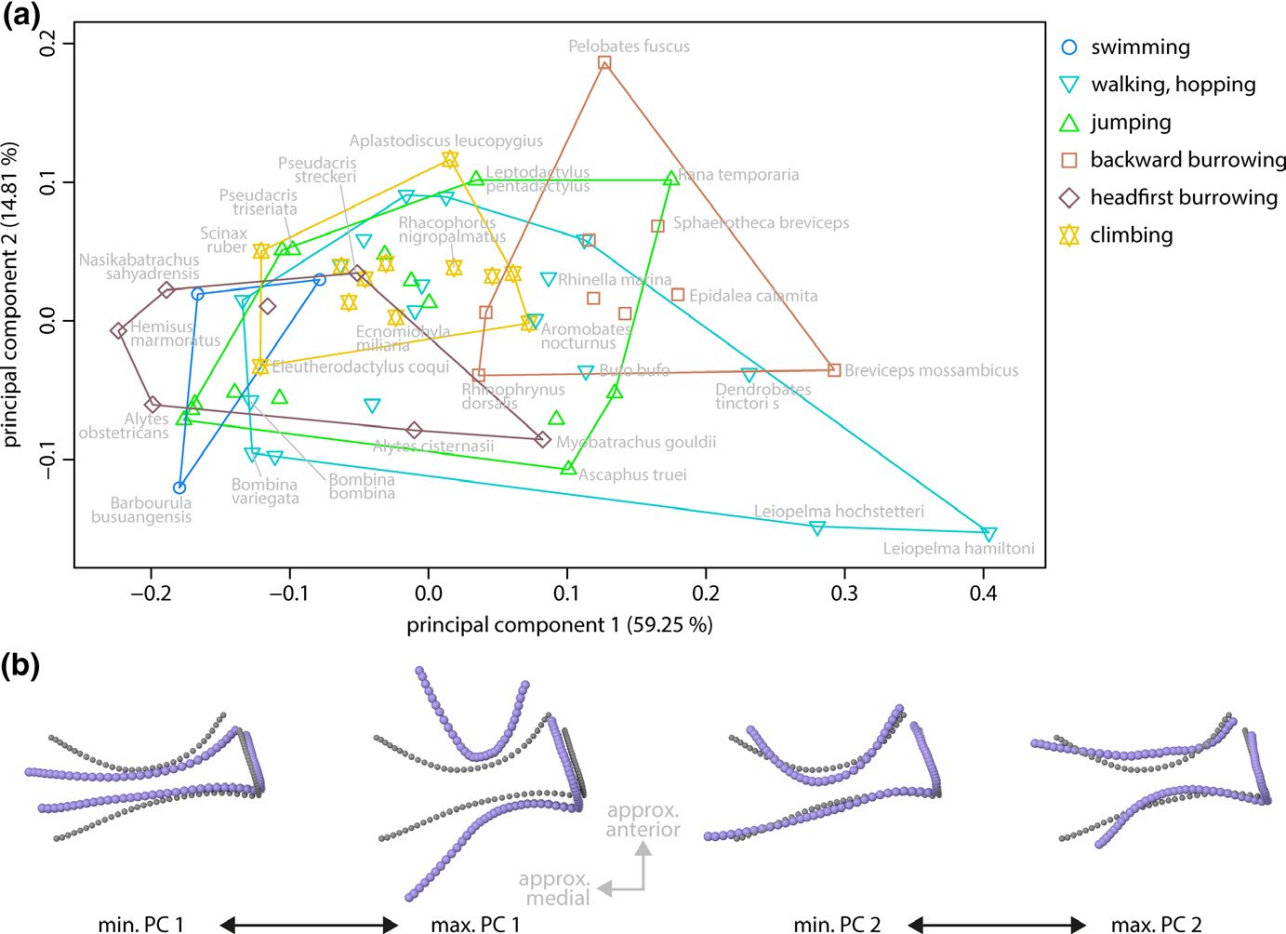
Principal component (PC) plot of species mean shapes of coracoids (landmark dataset iii) with extreme shapes along PCs. (a) PC plot of species mean shapes. (b) Extreme shapes of PC 1 in ventral view. (c) Extreme shapes of PC 2 in ventral view. Gray: mean shape; violet: extreme shape

Headfirst burrowing and swimming species differed from backward burrowing species along the first two PCs. There was no specific pattern with regard to group‐related shape differences along PC 3. The coracoid shapes mainly differed in their length (long axis, approx. medial‐lateral expansion) relative to their width (anterior–posterior expansion) in combination with different degrees of curvature of the anterior and posterior margin (Figure 8). These shape differences were associated with PC 1. The shape variation along PC 2 mainly represented differences in the curvature of the long axis in the anterior–posterior direction in combination with differences in the curvature of the anterior and posterior margins.

The phylogenetic signal in the species mean coracoid shape was significant (*K*_mult_ = 0.6355; *p *= .001), yet small compared to the phylogenetic signal in the overall pectoral girdle shape and the shape of the scapula. The effect of locomotor mode on coracoid shape was significant (*R* ^2^ = 0.25533; Table 6), and the pairwise comparison of locomotor group mean shapes (Table 4) showed that backward burrowing species significantly differed from all other locomotor groups, except for swimming species.

**TABLE 6:** Results of pMANOVA of species mean shapes of coracoids performed for respective fixed landmarks and semilandmarks (landmark dataset iii)

	*df*	SS	MS	*R* ^2^	*F*	*p*
Log. centroid size	1	0.0003308	0.00033077	0.02087	1.6416	.129
Locomotor mode	5	0.0040459	0.00080918	0.25533	4.0159	.001*
Log. centroid size:locomotor mode	5	0.0006368	0.00012736	0.04019	0.6321	.894
Residuals	52	0.0104776	0.00020149	0.66123		
Total	63	0.0158455				

Note

Asterisks (*) denote statistical significance.

### 3.4 Shapes of the cleithrum and clavicula

There were significant phylogenetic signals in the species mean shapes of the cleithrum (landmark dataset iv; *K*_mult_ = 0.5113; *p *= .001) and the clavicula (landmark dataset v; *K*_mult_ = 0.8752; *p *= .001). The pMANOVAs revealed no significant effects of locomotor group or log‐transformed centroid size and the principal components showed no clear pattern of separation of locomotor groups for any of the two landmark datasets (iv, v), although the first three (iv) or four (v) PCs were significant.

### 3.5 Muscle moment arms

The moment arms (Figure 4d–f) of the hypothetical muscles showed that the action of the muscles depended on the position of the humerus. The range of humerus positions (protraction–retraction) in which the posterior muscles contributed to retraction generally was largest in burrowing species. The moment arms of this muscle for retraction also were generally larger in burrowing species. The one exception to these observations was the backward burrowing species *Sphaerotheca breviceps* which showed comparably small moment arms for the posterior muscle during retraction and had a relatively small range of humerus positions in which this muscle contributed to retraction. The sternal muscle in this species, however, showed similar properties as the posterior muscles in the other burrowing species.

The properties of the other muscles showed no clear pattern of association with locomotor groups. Similar to the sternal muscle, the existence of a episternal muscle (if present) increased the moment arm for humerus protraction and widened the range of humerus positions for which the muscles contributed to protraction if compared to the anterior muscle of the respective species.

### 3.6 Finite element analysis of coracoids

Within each species, lowest mesh‐weighted arithmetic mean (mwam) von Mises stresses were observed if the coracoid was loaded along its long axis (scenario I; Figure 5). Highest stresses occurred if the posteromedial surface of the coracoid was pulled backward to simulate the potential effect of a m. sterno‐epicoracoideus or m. rectus abdominis (scenario V), and second‐highest stresses occurred if the same region was pushed forward to simulated potential forces transmitted by a sternum (scenario IV). Across species, the coracoid of *Breviceps mossambicus* experienced lowest mwam von Mises stresses under all loading scenarios. The coracoid of *Hemisus marmoratus* experienced highest mwam von Mises stresses under loading through the shoulder joint in lateromedial direction (scenario II) or by the hypothetical action of the posterior muscle (scenario III).

## 4 DISCUSSION

Our data indicate that the phylogenetic history, the size, and the locomotor behavior have significant effects on the shape of the pectoral girdle bones of anurans but the relative impact of these factors differs between bones. The most striking locomotor‐behavior‐related shape differences were observed between burrowing and nonburrowing species; those differences might be explained by a functional adaptation to the burrowing behavior and are possibly associated with trade‐offs. The shapes of the other locomotor groups differed less or even not at all and most groups showed remarkable within‐group shape diversity. Similarly shaped pectoral girdles provide the anatomical base for different locomotor behaviors, which indicates that the processes of many‐to‐one mapping (i.e., different morphologies can result in the same functional performance which might lead to a partial decoupling of morphological characters and function; Wainwright, Alfaro, Bolnick, & Hulsey, 2005) has acted during the evolution of the anuran pectoral girdle.

### 4.1 Modularity and phylogenetic signal

The observed differences in the relative impact of the considered factors (phylogeny, size, locomotion) on the shape of the distinct pectoral girdle bones might indicate some modularity within the pectoral girdle of anurans. This is supported by the statistical significance of the modularity test, although the result of this test should be interpreted with caution, as the test was performed on fixed landmarks and semilandmarks (Cardini, 2019).

At least some anatomical traits of anurans are influenced by their phylogenetic history; among these traits are the absolute and relative length of the hind limbs (Gomes, Rezende, Grizante, & Navas, 2009), the relative length of the tibiofibula and femur, their ratio, and the snout–vent length (Enriquez‐Urzelai et al., 2015), the relative length of the foreleg (Vidal‐García, Byrne, Roberts, & Keogh, 2014), and several other external body dimensions (Sherratt, Vidal‐García, Anstis, & Keogh, 2017). Our results are in line with these previous studies as the species mean shapes of the entire pectoral girdle and of its distinct bones showed a significant phylogenetic signal. There were differences, however, in the relative strength of the phylogenetic effect on the shapes of the single bones as indicated by different values of *K_mult_*. The species mean shapes of the scapulae resembled each other more than expected under a Brownian motion model (*K*_mult_ > 1), which implies that the phylogenetic history is the major factor in the evolution of the scapula shape. This is also supported by the observed differences in the shapes of the scapulae of non‐neobatrachian and neobatrachian species (Figures 1 and 7). The effects of size and locomotor mode, although statistically significant, seem to influence the scapula shape to a minor extent. In contrast, the observed phylogenetic signal in the species mean coracoid shape was comparably small and below the expectation under Brownian motion (*K*_mult_ < 1). This indicates that other factors (i.e., locomotion) besides phylogeny influence the evolution of the coracoid shape.

Among the factors considered herein, the phylogenetic relation seems to be the only factor to determine the shapes of the cleithrum and clavicula as the statistical analyses were insignificant for the factors size and locomotor group. But this might be an artifact caused by the GPA or pMANOVA, as the shape of each of these bones was analyzed using one curve of more or less colinear semilandmarks only. There might be an association of the shape of these bones with size or locomotion that was not detected by our analyses.

These observations allow the hypothesis that the evolution of the shape of the distinct pectoral girdle bones is driven by different primary factors, although they are part of the same complex. If so, this could indicate differences in the functional importance of these bones.

### 4.2 Adaptation of pectoral girdle shape to burrowing behavior

The most striking differences in the pectoral girdle shape were observed between burrowing and nonburrowing species (Figures 6 and 8; Table 4), which is in general accordance with previous studies that reported burrowing behavior to be associated with modifications of various anatomical structures (summarized in the introduction). The mean pectoral girdle shapes of backward and headfirst burrowing species significantly differ from one another and from other locomotor groups in one or more aspects (Table 4), indicating that the pectoral girdle bones of burrowing frogs may be specifically adapted to burrowing behavior. In particular, increased moment arms of the humerus retractor muscles (herein modeled as the posterior muscle) and widened ranges of humerus positions, for which this muscles acts as a retractor, were observed for most burrowing species if compared to nonburrowing species by musculoskeletal modeling (Figure 4e). This might be explained by specific biomechanical requirements linked to burrowing.

Emerson (1976) observed that specimens of the headfirst burrowing species *Hemisus marmoratus* moved the soil by forelimb retraction and that this motion was accompanied by a lateral force component. She assumed the enlarged retractor muscles and the elongated, posteriorly angled coracoids found in this species to be adaptations to the headfirst burrowing behavior. Our results indicate additional effects of the shape and orientation of the coracoid: The specific configuration of the coracoid shifted the origin of the posterior muscle backwards and thereby increased its moment arm across the shoulder joint, that is, its effectiveness (Sherman, Seth, & Delp, 2013) in humerus retraction if compared to other species (Figure 4). In addition, the posterior muscle functioned as a humerus retractor in a more anterior humerus position. Both these effects seem to be advantageous for headfirst burrowing and, thus, likely are adaptations to the burrowing behavior of *H. marmoratus*.

The finite element analyses revealed that the coracoid of *H. marmoratus* experienced comparable high mesh‐weighted arithmetic mean von Mises stresses if loading by the posterior muscle (scenario III) or by mediolateral compression (scenario II) was simulated (Figure 5c). This is somewhat surprising as both these loading scenarios seem ecologically relevant: The posterior muscle simulated the forces produced by the humerus retractor muscles, and there is a lateral force loading the pectoral girdle during headfirst digging (compare Emerson, 1976). The comparably high von Mises stress might be a trade‐off for the enlarged muscle moment arms across the shoulder joint caused by the elongation and specific orientation of the coracoid. It should be noted that the force of the posterior muscle was simulated for the humerus being orientated perpendicular to the sagittal plane; the observations of Emerson (1976) indicate that highest digging forces might occur in a more anterior humerus position. If so, the peak force imposed by the posterior muscle would be more aligned with the long axis of the coracoid, which in turn could result in smaller mean von Mises stress (also compare scenario I).

With regard to the pectoral girdle resisting to medially directed compression, it is noteworthy that the clavicula in *H. marmoratus* is angled rather perpendicular to the ventral midline, more robust, and enlarged medially (Figure 6b; also see Braus, 1919; Emerson, 1976). This shape and orientation somewhat resemble the configuration of the coracoid in some other species, and we hypothesize that, in *H. marmoratus*, the clavicula replaces the coracoid, for example, in transmitting and dissipating medially directed compressive forces through the shoulder joint. If this was true and the clavicula resisted most of the forces imposed by a medially directed compression, the mediolateral bending of the coracoid would be considerably reduced, which in turn would have led to smaller von Mises stresses in the coracoid. Such an effect was not observed in our simulations as we artificially removed the clavicula and the scapula, but the fusion of these two bones to the coracoid (Figure 6b) might be an indicator for their interaction in force transmission.

The specific clavicula configuration observed in *H. marmoratus* results in a small moment arm for the anterior muscle (Figure 4d) with regard to humerus protraction. Such small moment arms with regard to humerus protraction should be a disadvantage for headfirst burrowing as the retracted humerus needs to be moved forward for a new digging cycle. The bony episterum in *H. marmoratus* might have evolved to compensate for this disadvantage by expanding the area for muscle attachment anteriorly, which in turn leads to a larger moment arm across the shoulder joint (see episternal muscle in Figure 4d; also compare Trueb, 1973).

Large moment arms for the humerus retractor muscles seem to be a requirement for backward burrowing, too (compare Figure 4e), but the reason for this is not as obvious as for headfirst burrowing. To our knowledge, no detailed description of the function of the forelimbs (i.e., the forces acting on them) during backward burrowing does exist. The forelimbs are species‐specifically either used to stabilize the body (Emerson, 1976; Sanders & Davies, 1983) or to turn the body in the excavated hole (Sanders & Davies, 1983) during backward burrowing. Considering these functions, it might be hypothesized that the humerus retractor muscles mainly act to stabilize the shoulder joint while digging with the hind limbs, but this needs to be investigated in future studies.

It is remarkable that the coracoid of *Breviceps mossambicus* experienced lowest von Mises stresses in the finite element analyses (Figure 5d). Among the simulated loading scenarios, the resistance to lateral compression (scenario II) and to forces imposed by the humerus retractor muscles (scenario III) seem to be the most ecologically relevant, as backward digging is associated with a lateral force component (Emerson, 1976) and the retractor muscles likely are active during digging. The specific coracoid shape may thus be an adaptation to the backward burrowing behavior in *B. mossambicus* and comes at the cost of a small moment arm of the posterior muscle with regard to humerus retraction (Figure 4e). Analogous to the episternum in *H. marmoratus* (and other species), the pars ossea of the sternum in *B. mossambicus* might have evolved to compensate for this presumably disadvantageous moment arm (also compare Trueb, 1973). Cartilaginous episternal or sternal elements, as described for various species (e.g., Braus, 1919; Fürbringer, 1873; Trueb, 1973), were not considered herein. Yet, they might have a similar advantageous effect on muscle moment arms across the shoulder joint and should be included in future studies.

Two further observations support the hypothesis that the pectoral girdles of different species are adapted to their burrowing: *Alytes cisternasii* has been reported to be the faster and more efficient headfirst burrower if compared to the also headfirst burrowing *A. obstetricans* (Brown & Crespo, 2000). This coincides with the pectoral girdle shape of *A. obstetricans* being within the range of walking, hopping and jumping species, whereas the shape of *A. cisternasii* more resembles that of other headfirst burrowing species (Figure 6a). Thus, some anatomical specialization in the pectoral girdle of *A. cisternasii* might allow this species to perform better in burrowing. Despite the significant phylogenetic signal, the shape differences in the pectoral girdles of the jumping species *Pseudacris triseriata* and the headfirst burrowing species *P. streckeri* are comparably large with the latter more closely resembling the shape of other burrowing species (Figure 6a).

### 4.3 Walking, hopping, and jumping

In contrast to previous studies (see introduction for a summary), our analyses indicate that there is no specific pectoral girdle shape associated with either of these locomotor modes (Table 4) and, in particular, both locomotor groups do not differ in their mean pectoral girdle shape. Instead, walking, hopping and jumping species display a remarkable within‐group shape diversity in the pectoral girdle bones and their orientation to one another (Figures 6, 7, 8). It appears that differently shaped pectoral girdles are equally suited to fulfill the biomechanical requirements of jumping or walking, hopping.

### 4.4 Swimming and climbing

Swimming species significantly differed from headfirst burrowing species, as well as climbing from jumping species in the mean shapes of the scapulae only. These differences, although observed in the context of locomotor behavior, could be caused by the phylogenetic structure of the respective locomotor groups: The group of swimming species consisted of mostly non‐neobatrachians whereas the group of headfirst burrowing species consisted of non‐neobatrachians and neobatrachians (Figure 1). Given the strong phylogenetic signal in the scapula shape, this unequal phylogenetic pattern in locomotor group composition alone might have separated both groups in morphospace and there might be no true shape difference caused by differences in the locomotor behavior (also see the discussion of group dispersion across the phylogeny in Adams & Collyer, 2018).

The potential lack of a specific pectoral girdle shape within aquatic species might be explained by the fact that most anurans are good swimmers and likely have pectoral girdles that allow for an efficient aquatic locomotion. If so, the pectoral girdle shape of purely aquatic species would not differ much from nonaquatic species. An additional explanation for the nonspecific pectoral girdle shape of swimming anurans might be that the forelimbs are involved in swimming to only a minor extent (Abourachid & Green, 1999; Gillis & Biewener, 2000) and thus likely impose rather unspecific biomechanical requirements on the pectoral girdle. In addition, the effect of gravity is reduced in water (Zug, 1971) which would result in, among other things, minor forces acting on the pectoral girdle. Instead of being optimized for a high locomotor performance, the pectoral girdle of aquatic anuran species might be adapted to other ecologically relevant tasks like suction feeding (Cundall, Fernandez, & Irish, 2017). The morphological adaptation to swimming might have primarily occurred in other anatomical traits (Gómez & Lires, 2019; Laurent, 1964; Moen, 2019; Videler & Jorna, 1985).

Following the lines of argumentation above, it might be possible that there is no locomotor‐behavior‐related shape difference between jumping and climbing species, as the latter group consisted of neobatrachian species only, whereas the former additionally contained non‐neobatrachians. It is noteworthy that climbing evolved several times independently within the Neobatrachia only (Reilly & Jorgensen, 2011). Considering the phylogenetic distribution of arboreality, some specific anatomical novelties might have evolved in the last common ancestor of neobatrachian anurans and might have been necessary for the evolution of climbing behavior. The development of a fibrous epidermis with modified mucus glands on the finger and toe pads seems a promising candidate for such a novelty, as these specifications are not present in the non‐neobatrachian species *Ascaphus truei*, *Alytes obstetricans*, and *Scaphiopus holbrookii* (Noble & Jaeckle, 1928). In addition, those glands evolved before arboreality in certain anuran linages and were suggested to lead to climbing ability if combined with enlarged toe pads (Noble & Jaeckle, 1928). The lack of such a novelty might have constrained non‐neobatrachians from developing climbing behavior. Given that neobatrachians have comparably high scapulae and that climbing has evolved within neobatrachians only, these specific shapes seem to be associated with climbing although the true reason for the association likely is phylogenetic relatedness. All this is speculative at this stage and requires further investigation.

### 4.5 Many‐to‐one mapping and trade‐offs

The locomotor groups in our study showed a remarkable within‐group pectoral girdle shape diversity (Figures 6, 7, 8). Differently shaped pectoral girdles within a given locomotor group, thus, provide the anatomical base for similar locomotor behavior. This phenomenon of different forms allowing similar functions is known as many‐to‐one mapping (Wainwright et al., 2005) and, although not named as such, has previously been indicated for the anuran pectoral girdle. Arciferal and firmisternal pectoral girdles showed no considerable differences in patterns of deformation if compressively loaded through the shoulder joint (Emerson, 1984) and should thus be equally suited to accomplish tasks that require the resistance to lateral forces. Both girdle types, however, differ in the mechanism of how these forces are dissipated (Emerson, 1983; also see Figure 5). One additional example of many‐to‐one mapping has been observed in our study: Similar moment arms of the posterior muscle are produced by different pectoral girdle shapes in burrowing species (Figure 4). The coracoids in the respective girdles presumably accomplish different functions, namely either shifting the attachment area of the posterior muscle posteriorly or resisting mediolateral forces.

We observed few, if any, significant shape differences between swimming, jumping, climbing and walking, hopping species, and large regions of overlap of locomotor groups in morphospace (Figures 6, 7, 8). Similarly shaped pectoral girdles, thus, provide the anatomical base for different locomotor behaviors. This might be associated with trade‐offs imposed by conflicting biomechanical demands (Herrel, van Damme, Vanhooydonck, Zaaf, & Aerts, 2000). On the other hand, many‐to‐one mapping is thought to allow for the simultaneous optimization of multiple biomechanical properties (Wainwright, 2007; Wainwright et al., 2005), so that a given pectoral girdle shape might be equally adapted to several locomotor behaviors without functional trade‐offs.

Both, many‐to‐one mapping and trade‐offs, might have occurred during the evolution of the morphological diversity in anurans. For example, Moen (2019) observed many‐to‐one mapping in the relative hind limb length and relative hind limb muscle mass onto swimming and jumping performance. Neither trade‐offs nor coupled optimization between the independently evolved (Abourachid & Green, 1999; Astley, 2016) locomotor modes of swimming and jumping were observed for the hind limb anatomy of a semiaquatic frog (Nauwelaerts, Ramsay, & Aerts, 2007). Anurans with different pelvic and hind leg morphologies showed similar swimming abilities and that there was no trade‐off with jumping performance (Gal & Blake, 1987). These reports indicate many‐to‐one mapping (but see Robovská‐Havelkova et al., 2014 for a report of species with different ecologies showing different kinematic patterns of hind limb motion during swimming). A trade‐off has been reported between the maximum jumping distance and the jumping endurance with larger jumping distances being accompanied by an earlier onset of fatigue (Rand, 1952; Zug, 1978, 1985). Additionally, the relatively short legs of burrowing species are thought to be a trade‐off between efficient burrowing and jumping performance (Gomes et al., 2009). With regard to the anuran pectoral girdle, further studies are needed to analyze the biomechanical properties and resulting locomotor performances in order to assess which mechanisms, many‐to‐one mapping, trade‐offs, or both, acted during the evolution of this functional complex.

### 4.6 Potentially undetected adaptation of pectoral girdle shape to function

Despite our observations, there might be some functional adaptation of the pectoral girdle shape to more specific motion patterns than implied by our coarse definitions of walking, hopping, jumping, swimming, and climbing. Following Emerson (1979), we defined walking, hopping, and jumping locomotion based on the maximal leap length achieved by a given species. The length of a leap is determined during the initial phase of a jump by the amount of propulsive forces generated by the hind limbs (Hirano & Rome, 1984). If active at all, the forelimbs only raise the body and control the takeoff angle and do not contribute much to force generation (Akella & Gillis, 2011; Wang et al., 2014). This means that the pectoral girdle experiences comparably low forces during the initial phase and there might be no selective pressure for a specific girdle shape or function. Different landing behaviors have evolved within hopping or jumping anurans ranging from “belly flops” that do not involve the forelimbs, to coordinated landing during which the impact forces are transmitted and dissipated by the forelimbs and the pectoral girdle (Emerson, 1983; Essner, Suffian, Bishop, & Reilly, 2010; Griep et al., 2013; Reilly et al., 2016). Likewise, various landing patterns have been observed in an arboreal frog (Bijma, Gorb, & Kleinteich, 2016) and some climbing species are capable of parachuting or gliding (Oliver, 1951; also see Appendix S1: Tables A1, A2). It seems reasonable to assume that these different landing behaviors, as well as parachuting and gliding, are associated with different force patterns that act on the pectoral girdle and require specific skeletal and muscular geometries to be dissipated, particularly as landing force can be up to three times higher than the forces generated during takeoff (Nauwelaerts & Aerts, 2006).

The forelimbs of anurans are involved in other species‐specific behaviors besides locomotion as, for example, prey manipulation (Gray, O’Reilly, & Nishikawa, 1997) or wiping of the body surface (Blaylock, Ruibal, & Platt‐Aloia, 1976). The shapes of the pectoral girdle bones might be functionally adapted to these specific motion patterns and, given the significant phylogenetic signal and the potential effects of many‐to‐one mapping, might occur on a smaller scale within closely related groups. These hypotheses were not tested herein.

The literature record on anuran behavior and our definition of locomotor groups might be insufficient to fully represent the behavior of at least some species. For example, the backward burrowing species *Rhinophrynus dorsalis* is hypothesized to be capable of headfirst burrowing (Trueb & Gans, 1983). *Aplastodiscus leucopygius* is an arboreal species (Ferreira et al., 2008; Haddad & Sawaya, 2000), but at least the males have been observed to use their heads for the construction of subterranean nests that serve for egg deposition (Haddad & Sawaya, 2000). Both these species are located within or close to the region acclaimed by the group of headfirst burrowing anurans in morphospaces (Figures 6, 7, 8). Headfirst burrowing might thus require a pectoral girdle with specific biomechanical properties (potentially realized by different morphologies) and there, thus, might be adaptations to locomotor behavior that were not detected by our approach.

### 4.7 Limitations and future perspectives

Most anuran species in our sample were represented by one specimen only, and shape analyses were performed on the mean shapes of species. We did not consider sexual dimorphism, although this phenomenon has been reported for the humerus in some species (Lee, 2001; Padhye, Jadhav, Sulakhe, & Dahanukar, 2015; Petrović, Vukov, & Kolarov, 2017) and some muscles originating from the pectoral girdle (Emerson, 1990; Lee, 2001; Oka, Ohtani, Satou, & Ueda, 1984). Sexual dimorphism may, thus, be expected to occur in the pectoral girdle bones, too. Nevertheless, we expect these limitations to have a minor effect on our results, as the shapes of all landmark sets of a given species lay mostly within the same respective locomotor group in morphospace or expanded the region claimed by the locomotor group toward more extreme shapes without enlarging the overlap with other locomotor groups (Figure 6). Yet, sexual dimorphism and intraspecific variability in the shape of the anuran pectoral girdle bones would be interesting topics for future studies and, if combined with behavioral and biomechanical analyses, could shed light on the functional and ecological consequences of shape differences.

Muscle moment arms were simulated using a simplified humerus with all hypothetical muscles inserting at the same point in order to assess the effects of different pectoral girdle geometries independent of other factors. As Emerson (1991) argued, the length of the humerus and the location of the muscle attachments along its length influence the resulting mechanical advantage. Thus, our analysis explored only one aspect among the factors determining the biomechanical properties of the shoulder joint. Assessing the combined effects of pectoral girdle and humerus shape, as well as the consideration of species‐specific muscle configurations, could provide further insight into the functionality of this complex and explain its evolution.

## CONFLICT OF INTEREST

The authors declare that there is no conflict of interest.

## AUTHOR CONTRIBUTION

**Karolin Engelkes:** Conceptualization (lead); Data curation (equal); Formal analysis (lead); Funding acquisition (supporting); Investigation (lead); Methodology (lead); Project administration (lead); Software (lead); Supervision (equal); Validation (lead); Visualization (lead); Writing‐original draft (lead); Writing‐review & editing (equal). **Lena Kath:** Data curation (equal); Investigation (supporting); Writing‐review & editing (supporting). **Thomas Kleinteich:** Investigation (supporting); Resources (supporting); Writing‐review & editing (supporting). **Jörg Hammel:** Investigation (supporting); Resources (supporting); Writing‐review & editing (supporting). **André Beerlink:** Investigation (supporting); Resources (supporting); Writing‐review & editing (supporting). **Alexander Haas:** Data curation (equal); Funding acquisition (lead); Supervision (equal); Writing‐original draft (supporting); Writing‐review & editing (equal).

## Supporting information

Fig S1

Appendix S1

## Data Availability

CT volumes can be downloaded from https://www.fdr.uni‐hamburg.de/search?page=1&size=20&q=keywords:%22pectoral%20girdle%20morphometrics%20project%22; DOI numbers are provided in Appendix S1: Table A1.
