# Supplementary figures and images for: Ecomorphology of the pectoral girdle in anurans (Amphibia, Anura): Shape diversity and biomechanical considerations
Source: Ecol Evol. 2020 Sep 17;10(20):11467–87. doi: 10.1002/ece3.6784 (PMC7593145; doi:10.1002/ece3.6784)

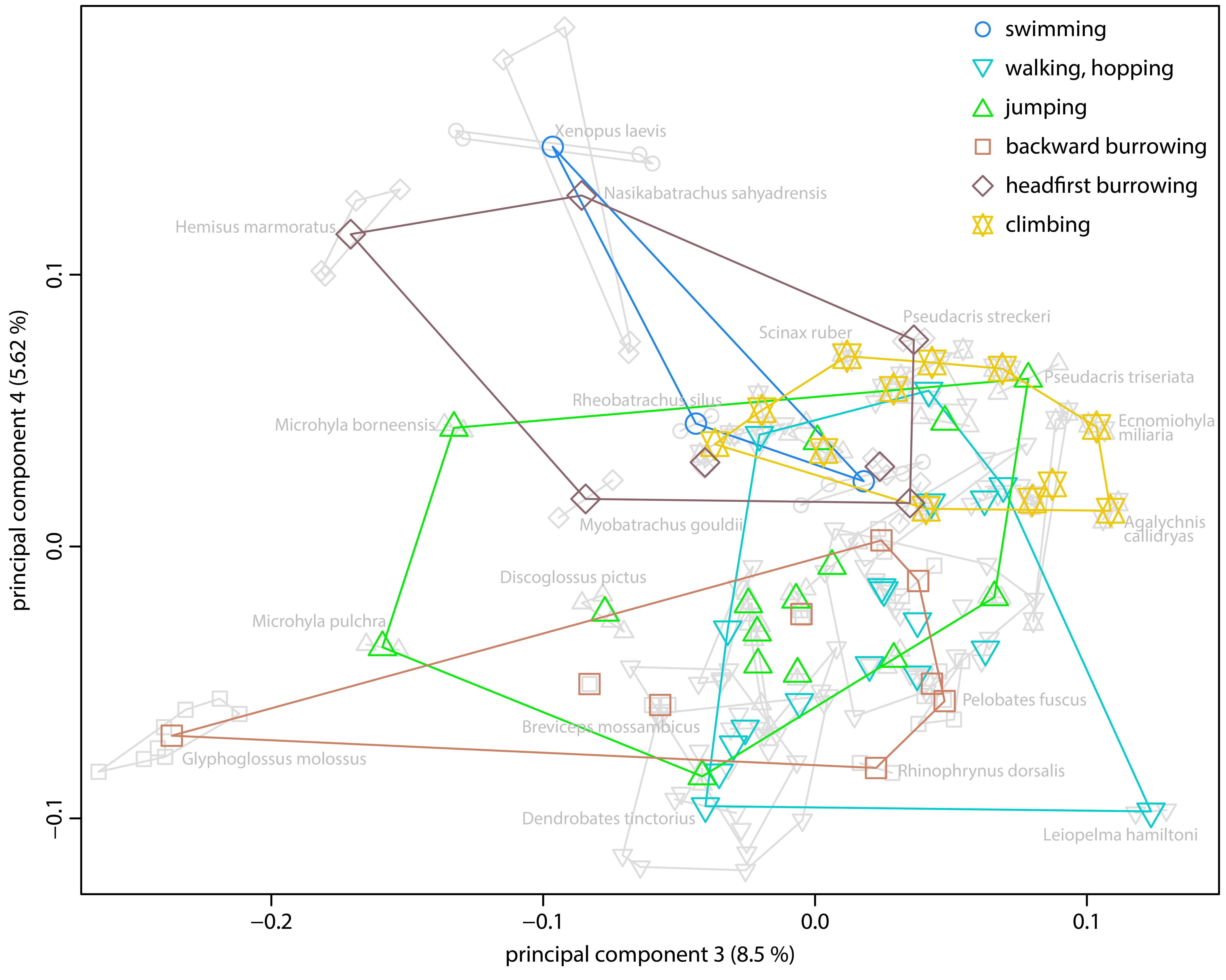

Supplement: Supplementary file 1 — Fig S1 [file ECE3-10-11467-s001.tif]
