# Supplementary material for: Ecomorphology of the pectoral girdle in anurans (Amphibia, Anura): Shape diversity and biomechanical considerations
Source: Ecol Evol. 2020 Sep 17;10(20):11467–87. doi: 10.1002/ece3.6784 (PMC7593145; doi:10.1002/ece3.6784)
Supplement: Supplementary file 2 — Appendix S1 [file ECE3-10-11467-s002.docx]

# Appendix

**FIGURE A1** Plot of principal components 3 and 4 of overall species mean shapes of pectoral girdle bones (landmark dataset i). Grey points illustrate single landmark configurations used to calculate the species mean shapes.

**TABLE A****1** Specimens, locomotor modes, and CT scanning parameter. Museum abbreviations: AMNH: American Museum of Natural History, New York; CAS: California Academy of Sciences, San Francisco; Erfurt: Naturkundemuseum Erfurt, Erfurt; FMNH: Field Museum of Natural History, Chicago; USMN: National Museum of Natural History, Washington, D.C.; ZMB: Museum für Naturkunde, Berlin; ZMH: Zoologisches Museum Hamburg, Hamburg; ZSM: Zoologische Staatssammlung, Munich.

| **Species** | **Locomotor mode** | **Reference** | **Catalogue number** | **CT scanning parameters** [**doi number]** | **Remark** |
| --- | --- | --- | --- | --- | --- |
| *Afrixalus dorsalis* (Peters, 1875) | climbing | Jorgensen & Reilly 2013 | ZMB 71328 | Skyscan1172; 52 kV; 188 µA; filter: Al 0.5 mm; voxel size: 13.33 µm  [10.25592/uhhfdm.1142] |  |
| *Alytes obstetricans* (Laurenti, 1768) | headfirst burrowing | Brown & Crespo 2000 | ZMH A12442 | Skyscan1172; 49 kV; 200 µA; filter: Al 0.5 mm; voxel size: 21.34 µm  [10.25592/uhhfdm.1144] |  |
| *Amietia angolensis* (Bocage, 1866) | jumping | Laurent 1964; Emerson 1976 | ZMH A07325 | Skyscan1172; 55 kV; 179 µA; filter: Al 0.5 mm; voxel size: 22.40 µm  [10.25592/uhhfdm.1146] |  |
| *Aplastodiscus leucopygius* (Cruz and Peixoto, 1985) | climbing | Haddad & Sawaya 2000; Ferreira *et al.* 2008; Berneck, Segalla & Haddad 2017 | USMN 208405 | Skyscan1172; 52 kV; 188 µA; filter: Al 0.5 mm; voxel size: 21.08 µm  [10.25592/uhhfdm.1148] | Headfirst burrowing in males for nest construction |
| *Aromobates nocturnus* Myers, Paolillo-O., and Daly, 1991 | walking, hopping | Myers, CharleMyers, Charles W., Paolillo O., Daly, John W. s W. & Daly 1991 | AMNH A130017 | Phoenix Nanotom S; 100 kV; 70 µA; no filter; voxel size: 30.00 µm  [10.25592/uhhfdm.1152] |  |
| *Barbourula busuangensis* Taylor and Noble, 1924 | swimming | Myers 1943 | CAS-SUA 21240 | Skyscan1172; 100 kV; 100 µA; filter: Al+Cu; voxel size: 26.68 µm  [10.25592/uhhfdm.1156] |  |
|  |  |  | CAS-SUA 21247 | Skyscan1172; 100 kV; 100 µA; filter: Al+Cu; voxel size: 26.68 µm  [10.25592/uhhfdm.1294] |  |
| *Bombina bombina* (Linnaeus, 1761) | walking, hopping | Zug 1978; Cevik, Baskale & Kaya 2008 | ZMH A05110 | YXLON FF35 CT; 100 kV; 120 µA; no filter; voxel size: 22.7455 µm  [10.25592/uhhfdm.1162] | Scans from Engelkes *et al.* 2019 |
|  |  |  | ZMH A05383 | YXLON FF35 CT; 100 kV; 120 µA; no filter; voxel size: 22.75 µm  [10.25592/uhhfdm.1164] |  |
|  |  |  | ZMH A05617 | YXLON FF20 CT; 80 kV; 80 µA; no filter; voxel size: 25.84 µm  [10.25592/uhhfdm.1166] |  |
|  |  |  | ZMH A09674 | Skyscan1172; 49 kV; 200 µA; filter: Al 0.5 mm; voxel size: 21.34 µm  [10.25592/uhhfdm.1168] | Only left pectoral girdle half used; Scan from Engelkes *et al.* 2019 |
| *Bombina maxima* (Boulenger, 1905) | walking, hopping | Jorgensen & Reilly 2013; Mai, Yu & Liao 2019 | ZMH A05082 | Skyscan1172; 70 kV; 139 µA; filter: Al+Cu; 26.68 µm  [10.25592/uhhfdm.1170] |  |
| *Bombina orientalis* (Boulenger, 1890) | walking, hopping | Emerson 1979 | ZMH A05672 | YXLON FF35 CT; 100 kV; 120 µA; no filter; voxel size: 30.33 µm  [10.25592/uhhfdm.1172] | Scans from Engelkes *et al.* 2019 |
|  |  |  | ZMH A05676 | Skyscan1172; 100 kV; 100 µA; filter: Al 0.5 mm; voxel size 21.34 µm  [10.25592/uhhfdm.1174] |  |
|  |  |  | ZMH A05677 | YXLON FF35 CT; 100 kV; 120 µA; no filter; voxel size: 22.75 µm  [10.25592/uhhfdm.1176] |  |
|  |  |  | ZMH A05678 | YXLON FF35 CT; 100 kV; 120 µA; no filter; voxel size: 22.75 µm  [10.25592/uhhfdm.1182] |  |
|  |  |  | ZMH A05681 | YXLON FF35 CT; 100 kV; 120 µA; no filter; voxel size: 22.75 µm  [10.25592/uhhfdm.1178] | Scans from Engelkes *et al.* 2019 |
|  |  |  | ZMH A05682 | YXLON FF35 CT; 100 kV; 120 µA; no filter; voxel size: 22.75 µm  [10.25592/uhhfdm.1180] |  |
| *Bombina variegata* (Linnaeus, 1758) | walking, hopping | pers. obs. AH | ZMH A06234 | Skyscan1172; 49 kV; 200 µA; filter: Al 0.5 mm; voxel size: 17.60 µm  [10.25592/uhhfdm.1184] | Only left pectoral girdle half used |
| *Breviceps mossambicus* Peters, 1854 | backward burrowing | Poynton 1982 | ZMB 83246 | Skyscan1172; 55 kV; 181 µA; filter: Al 0.5 mm; voxel size: 16.54 µm  [10.25592/uhhfdm.1186] |  |
| *Bufo bufo* (Linnaeus, 1758) | walking, hopping | Zug 1978; Enriquez-Urzelai, Montori, Llorente & Kaliontzopoulou 2015 | ZMH A04660 | YXLON FF 35 CT; 70 kV; 120 µA; no filter; voxel size: 25.8 µm  [10.25592/uhhfdm.1188] |  |
|  |  |  | ZMH A04680 | Phoenix Nanotom M; 120 kV; 400 µA; no filter; voxel size: 63.41 µm  [10.25592/uhhfdm.1190] | Only right pectoral girdle half used |
|  |  |  | ZMH A04682 | Phoenix Nanotom M; 120 kV; 400 µA; no filter; voxel size: 63.41 µm  [10.25592/uhhfdm.1192] |  |
|  |  |  | ZMH A04708 | Phoenix Nanotom M; 120 kV; 400 µA; no filter; voxel size: 63.41µm  [10.25592/uhhfdm.1194] |  |
|  |  |  | ZMH A04709 | Phoenix Nanotom M; 120 kV; 400 µA; no filter; voxel size: 63.41 µm  [10.25592/uhhfdm.1198] |  |
|  |  |  | ZMH A04717 | Phoenix Nanotom M; 120 kV; 400 µA; no filter; voxel size: 63.41 µm  [10.25592/uhhfdm.1200] |  |
| *Ceratophrys aurita* (Raddi, 1823) | backward burrowing | Nomura, Rossa-Feres & Langeani 2009; Natale *et al.* 2011 | ZMH A01393 | Phoenix v\|tome\|x L 450; 170 kV; 500 µA; no filter; voxel size: 106.01 µm  [10.25592/uhhfdm.1204] |  |
| *Crossodactylus caramaschii* Bastos and Pombal, 1995 | headfirst burrowing | Nomura, Rossa-Feres & Langeani 2009 | USMN 318234 | Skyscan1172; 49 kV; 200 µA; filter: Al 0.5 mm; voxel size: 11.87 µm  [10.25592/uhhfdm.1206] |  |
| *Dendrobates tinctorius* (Cuvier, 1797) | walking, hopping | Emerson 1979 | ZMH A12904 | Skyscan1172; 52 kV; 181 µA; filter: Al 0.5 mm; voxel size: 20.00 µm  [10.25592/uhhfdm.1208] |  |
| *Discoglossus montalentii* Lanza, Nascetti, Capula, and Bullini, 1984 | jumping | pers. obs. AH | ZSM 1299/2006 | Skyscan1172; 52 kV; 181 µA; filter: Al 0.5 mm; voxel size: 26.68 µm  [10.25592/uhhfdm.1212] |  |
|  |  |  | ZSM 1300/2006 | Skyscan1172; 60 kV; 165 µA; filter: Al+Cu; voxel size: 21.08 µm  [10.25592/uhhfdm.1214] |  |
| *Discoglossus pictus* Otth, 1837 | jumping | Emerson 1979 | ZSM 933/2010 | Skyscan1172; 49 kV; 200 µA; filter: Al 0.5 mm; voxel size: 23.20 µm  [10.25592/uhhfdm.1216] |  |
|  |  |  | ZSM 937/2010 | Skyscan1172; 55 kV; 179 µA; filter: Al 0.5 mm; voxel size: 26.68 µm  [10.25592/uhhfdm.1218] |  |
| *Discoglossus scovazzi* Camerano, 1878 | jumping | [based on other *Discoglossus* species] | ZMH A15451 | Skyscan1172; 49 kV; 200 µA; filter: Al 0.5 mm; voxel size: 21.34 µm  [10.25592/uhhfdm.1220] |  |
| *Epidalea calamita* (Laurenti, 1768) | backward burrowing | Emerson 1976, 1979 | ZMH A06868 | Skyscan1172; 100 kV; 100 µA; filter: Al 0.5 mm; voxel size: 22.94 µm  [10.25592/uhhfdm.1292] |  |
| *Fejervarya limnocharis* (Gravenhorst, 1829) | jumping | pers. obs. AH | ZMH A05523 | Skyscan1172; 100 kV; 100 µA; filter: Al 0.5 mm; voxel size: 21.34 µm  [10.25592/uhhfdm.1224] |  |
| *Gastrotheca riobambae* (Fowler, 1913) | climbing | Hertwig & Sinsch 1995 | CAS 119027 | Skyscan1172; 49 kV; 200 µA; filter: Al 0.5 mm; voxel size: 26.68 µm  [10.25592/uhhfdm.1226] |  |
| *Glyphoglossus molossus* Günther, 1869 | backward burrowing | Emerson 1976 | Erfurt A 1815/11 | Phoenix Nanotom M; 120 kV; 400 µA; no filter; voxel size: 46.08 µm  [10.25592/uhhfdm.1228] |  |
|  |  |  | Erfurt A 1818/11 | Phoenix Nanotom M; 120 kV; 400 µA; no filter; voxel size: 46.08 µm  [10.25592/uhhfdm.1230] |  |
|  |  |  | Erfurt A 1819/11 | Phoenix Nanotom M; 120 kV; 400 µA; no filter; voxel size: 46.08 µm  [10.25592/uhhfdm.1232] |  |
|  |  |  | Erfurt A 2186/15 | Skyscan1172; 100 kV; 100 µA; filter: Al+Cu; voxel size: 26.68 µm  [10.25592/uhhfdm.1234] |  |
| *Hemisus marmoratus* (Peters, 1854) | headfirst burrowing | Emerson 1976 | ZMB 79852 | Skyscan1172; 55 kV; 181 µA; filter: Al 0.5 mm; voxel size: 21.34 µm  [10.25592/uhhfdm.1236] |  |
|  |  |  | ZMH A06757 | Skyscan1172; 49 kV; 200 µA; filter: Al 0.5 mm; voxel size: 18.14 µm  [10.25592/uhhfdm.1238] |  |
| *Hyla arborea* (Linnaeus, 1758) | climbing | pers. obs.; Cott 1926 | ZMH A06468 | Phoenix Nanotom S; 60 kV; 150 µA; no filter; voxel size: 23.23 µm  [10.25592/uhhfdm.1240] | Only right pectoral girdle half used |
| *Hyperolius parallelus* Günther, 1858 | climbing | [Channing 2001 for the genus *Hyperolius*] | ZMH A09562 | Skyscan1172; 55 kV; 125 µA; filter: Al 0.5 mm; voxel size: 16.54 µm  [10.25592/uhhfdm.1242] |  |
| *Kaloula pulchra* Gray, 1831 | backward burrowing | Emerson 1976 | CAS 230419 | Skyscan1172; 49 kV; 200 µA; filter: Al 0.5 mm; voxel size: 18.68 µm  [10.25592/uhhfdm.1244] | Only left pectoral girdle half used |
| *Kassina senegalensis* (Duméril and Bibron, 1841) | walking, hopping | Emerson 1979 | ZMB 75810 | Skyscan1172; 52 kV; 188 µA; filter: Al 0.5 mm; voxel size: 19.74 µm  [10.25592/uhhfdm.1246] |  |
|  |  |  | ZMH A07354 | Skyscan1172; 49 kV; 200 µA; filter: Al 0.5 mm; voxel size: 17.87 µm  [10.25592/uhhfdm.1248] |  |
| *Leiopelma hochstetteri* Fitzinger, 1861 | walking, hopping | Worthy 1987 | CAS-SUA 9609 | Skyscan1172; 70 kV; 139 µA; filter: Al+Cu; voxel size: 20.27 µm  [10.25592/uhhfdm.1250] |  |
| *Leptobrachella mjobergi* Smith, 1925 | walking, hopping | Hennigan 2013 | ZMH A11518 | Skyscan1172; 51 kV; 194 µA; filter: Al 0.5 mm; voxel size: 13.33 µm  [10.25592/uhhfdm.1252] |  |
| *Leptodactylus pentadactylus* (Laurenti, 1768 | jumping | Emerson 1979 | ZMH A02559 | Phoenix v\|tome\|x L 450; 170 kV; 500 µA; no filter; voxel size: 91.40 µm  [10.25592/uhhfdm.1254] |  |
| *Microhyla nepenthicola* Das and Haas, 2010 | jumping | pers. obs. AH | ZMH A11645 | YXLON FF20 CT; 60 kV; 110 µA; no filter; voxel size: 12.69 µm  [10.25592/uhhfdm.1256] |  |
| *Microhyla pulchra* (Hallowell, 1861) | jumping | Emerson 1976 | USMN 278542 | Skyscan1172; 49 kV; 200 µA; filter: Al 0.5 mm; voxel size: 13.33 µm  [10.25592/uhhfdm.1258] |  |
| *Occidozyga baluensis* (Boulenger, 1896) | walking, hopping | pers. obs. AH | ZMH A10454 | Skyscan1172; 51 kV; 194 µA; filter: Al 0.5 mm; voxel size: 13.33 µm  [10.25592/uhhfdm.1260] |  |
| *Oreobates quixensis* Jiménez de la Espada, 1872 | jumping | Jorgensen & Reilly 2013 | AMNH A94687 | Skyscan1172; 100 kV; 100 µA; filter: Al 0.5 mm; voxel size: 26.68 µm  [10.25592/uhhfdm.1262] |  |
| *Pelobates fuscus* (Laurenti, 1768) | backward burrowing | Savage 1942 | ZMH A07151 | Skyscan1172; 70 kV; 139 µA; filter: Al+Cu; voxel size: 26.68 µm  [10.25592/uhhfdm.1266] |  |
| *Pelodytes punctatus* (Daudin, 1802) | jumping | Enriquez-Urzelai, Montori, Llorente & Kaliontzopoulou 2015 | ZMH A07281 | Skyscan1172; 55 kV; 165 µA; filter: Al 0.5 mm; voxel size: 21.34 µm  [10.25592/uhhfdm.1268] |  |
| *Pleurodema bibroni* Tschudi, 1838 | walking, hopping | Jorgensen & Reilly 2013 | FMNH 132507 | Skyscan1172; 55 kV; 181 µA; filter: Al 0.5 mm; voxel size: 21.34 µm  [10.25592/uhhfdm.1270] |  |
| *Pseudacris streckeri* Wright and Wright, 1933 | headfirst burrowing | Brown, Jackson & Brown 1972 | AMNH A184936 | Skyscan1172; 55 kV; 179 µA; filter: Al 0.5 mm; voxel size: 21.34 µm  [10.25592/uhhfdm.1272] |  |
| *Pseudacris triseriata* (Wied-Neuwied, 1838) | jumping | Emerson 1979 | CAS 188145 | Skyscan1172; 49 kV; 200 µA; filter: Al 0.5 mm; voxel size: 18.68 µm  [10.25592/uhhfdm.1274] |  |
| *Rana temporaria* Linnaeus, 1758 | jumping | pers. obs. | ZMH A11310 | Skyscan1172; 100 kV; 55 µA; no filter; voxel size: 26.18 µm  [10.25592/uhhfdm.1278] |  |
| *Rentapia hosii* (Boulenger, 1892) | climbing | pers. obs. AH | FMNH 244892 | Skyscan1172; 54 kV; 185 µA; filter: Al 0.5 mm; voxel size: 26.68 µm  [10.25592/uhhfdm.1264] |  |
| *Rhacophorus nigropalmatus* Boulenger, 1895 | climbing | Emerson & Koehl 1990 | ZMH A10414 | YXLON FF35 CT; 60 kV; 160 µA; no filter; voxel size: 20.02 µm  [10.25592/uhhfdm.1282] | Gliding |
| *Rhinella marina* (Linnaeus, 1758) | walking, hopping | Emerson 1979 | ZMH A01033 | Phoenix v\|tome\|x L 450; 170 kV; 500 µA; no filter; voxel size: 85.74 µm  [10.25592/uhhfdm.1284] |  |
| *Rhinoderma darwinii* Duméril and Bibron, 1841 | jumping | Emerson 1979 | ZMH A10873 | Skyscan1172; 55 kV; 181 µA; filter: Al 0.5 mm; voxel size: 13.33 µm  [10.25592/uhhfdm.1286] |  |
| *Rhinophrynus dorsalis* Duméril and Bibron, 1841 | backward burrowing | Trueb & Gans 1983 | CAS 71767 | Skyscan1172; 70 kV; 139 µA; filter: Al+Cu; voxel size: 21.60 µm  [10.25592/uhhfdm.1288] |  |
| *Scinax ruber* (Laurenti, 1768) | climbing | Pauly, Bernal & Taylor 2005 | ZMH A02098 | Skyscan1172; 51 kV; 192 µA; filter: Al 0.5 mm; voxel size: 21.08 µm  [10.25592/uhhfdm.1290] | Parachuting |
| *Xenopus laevis* (Daudin, 1802) | swimming | Emerson 1979 | ZMH A02374 | Skyscan1172; 100 kV; 100 µA; filter: Al 0.5 mm; voxel size: 26.68 µm  [10.25592/uhhfdm.1296] |  |
| *Zhangixalus prominanus* (Smith, 1924) | climbing | Shahrudin 2017 | [one of the specimens in Barnes, Baum, Peisker & Gorb 2013] | Skyscan1172; 49 kV; 200 µA; filter: Al 0.5 mm; voxel size: 26.68 µm [unpublished] |  |

**TABLE A****2** Specimens, locomotor modes, and MorphoSource media number. Museum abbreviations: CAS: California Academy of Sciences, San Francisco; CES: Centre for Ecological Science, Bangalore; CM: Carnegie Museum of Natural History, Pittsburgh; Erfurt: Naturkundemuseum Erfurt, Erfurt; FMNH: Field Museum of Natural History, Chicago; KUH: University of Kansas Biodiversity Institute, Lawrence; MCZ: Museum of Comparative Zoology, Cambridge; UF: Florida Museum of Natural History, Gainesville.

| **Species** | **Locomotor mode** | **Reference** | **Catalogue number** | **MorphoSource media number and URL** | **Remark** |
| --- | --- | --- | --- | --- | --- |
| *Agalychnis callidryas* (Cope, 1862) | climbing | Roberts 1994 | CAS Herp 146957 | M25577-50012, http://www.morphosource.org/Detail/MediaDetail/Show/media_id/25577 | Parachuting |
| *Alytes cisternasii* Boscá, 1879 | headfirst burrowing | Brown & Crespo 2000 | MCZ A-3494 | M35795-65967, http://www.morphosource.org/Detail/MediaDetail/Show/media_id/35795 |  |
| *Ascaphus truei* Stejneger, 1899 | jumping | Emerson 1979 | UF Herp 80664 | M8805-11256, http://www.morphosource.org/Detail/MediaDetail/Show/media_id/8805, doi:10.17602/M2/M11256 |  |
| *Bombina maxima* (Boulenger, 1905) | walking, hopping | Jorgensen & Reilly 2013; Mai, Yu & Liao 2019 | UF Herp 96648 | M9207-23561, http://www.morphosource.org/Detail/MediaDetail/Show/media_id/9207, doi:10.17602/M2/M23561 |  |
| *Ecnomiohyla miliaria* (Cope, 1886) | climbing | amphibiaweb.org (6^th^ February 2020) | UF Herp 137208 | M25112-49249, http://www.morphosource.org/Detail/MediaDetail/Show/media_id/25112 | Gliding |
| *Eleutherodactylus coqui* Thomas, 1966 | climbing | Stewart 1985 | UF Herp 21290 | M24647-48540, http://www.morphosource.org/Detail/MediaDetail/Show/media_id/24647, doi:10.17602/M2/M48540 | Parachuting |
| *Epipedobates tricolor* (Boulenger, 1899) | jumping | Jorgensen & Reilly 2013 | KUH 219763 | M24625-48513, http://www.morphosource.org/Detail/MediaDetail/Show/media_id/24625 |  |
|  |  |  | UF Herp 83888 | M24980-49096, http://www.morphosource.org/Detail/MediaDetail/Show/media_id/24980 |  |
| *Eupsophus roseus* (Duméril and Bibron, 1841) | walking, hopping | Meserve & Jaksic 1991 | CM Herp 57175 | M18659-35357, http://www.morphosource.org/Detail/MediaDetail/Show/media_id/18659 |  |
|  |  |  | CM Herp 63926 | M12692-23439, http://www.morphosource.org/Detail/MediaDetail/Show/media_id/12692, doi:10.17602/M2/M23439 |  |
| *Gastrotheca riobambae* (Fowler, 1913) | climbing | Hertwig & Sinsch 1995 | UF Herp 98224 | M28917-55509, http://www.morphosource.org/Detail/MediaDetail/Show/media_id/28917 |  |
| *Leiopelma hamiltoni* McCulloch, 1919 | walking, hopping | Worthy 1987 | CAS Herp 53931 | M13874-24351, http://www.morphosource.org/Detail/MediaDetail/Show/media_id/13874, doi:10.17602/M2/M24351 |  |
| *Leptobrachella mjobergi* Smith, 1925 | walking, hopping | Hennigan 2013 | FMNH 273699 | M23544-46055, http://www.morphosource.org/Detail/MediaDetail/Show/media_id/23544 |  |
| *Leptobrachium hasseltii* Tschudi, 1838 | walking, hopping | Hennigan 2013 | UF Herp 61841 | M10832-16288, http://www.morphosource.org/Detail/MediaDetail/Show/media_id/10832, doi:10.17602/M2/M16288 |  |
| *Megophrys stejnegeri* Taylor, 1920 | walking, hopping | Hennigan 2013 | KUH 321429 | M22890-44409, http://www.morphosource.org/Detail/MediaDetail/Show/media_id/22890 |  |
| *Mixophyes fasciolatus* Günther, 1864 | walking, hopping | Littlejohn, Roberts, Watson & Davies 1993; Jorgensen & Reilly 2013 | CAS Herp 82050 | M23916-47036, http://www.morphosource.org/Detail/MediaDetail/Show/media_id/23916 |  |
| *Myobatrachus gouldii* (Gray, 1841) | backward burrowing | Emerson 1976 | MCZ A 139543 | M25636-50088, http://www.morphosource.org/Detail/MediaDetail/Show/media_id/25636, doi:10.17602/M2/M50088 |  |
| *Nasikabatrachus sahyadrensis* Biju and Bossuyt, 2003 | headfirst burrowing | Senevirathne *et al.* 2016 | CES F 203 | M12152-19736, http://www.morphosource.org/Detail/MediaDetail/Show/media_id/12152 |  |
|  |  |  | CES F 877 | M12185-19838, http://www.morphosource.org/Detail/MediaDetail/Show/media_id/12185 |  |
| *Occidozyga baluensis* (Boulenger, 1896) | walking, hopping | pers. obs. AH | KUH 155619 | M25762-50252, http://www.morphosource.org/Detail/MediaDetail/Show/media_id/25762 |  |
| *Pelobates fuscus* (Laurenti, 1768) | backward burrowing | Savage 1942 | UF Herp 36935 | M25191-49377, http://www.morphosource.org/Detail/MediaDetail/Show/media_id/25191, doi:10.17602/M2/M49377 |  |
| *Pyxicephalus adspersus* Tschudi, 1838 | backward burrowing | Loveridge & Withers 1981; pers. obs. AH | UF Herp 92094 | M25376-49707, http://www.morphosource.org/Detail/MediaDetail/Show/media_id/25376 |  |
| *Rheobatrachus silus* Liem, 1973 | swimming | Liem 1973; Littlejohn, Roberts, Watson & Davies 1993 | CAS Herp 153753 | M23917-47037, http://www.morphosource.org/Detail/MediaDetail/Show/media_id/23917 |  |
| *Sphaerotheca breviceps* (Schneider, 1799) | backward burrowing | Nomura, Rossa-Feres & Langeani 2009 | UF Herp 20069 | M24970-49086, http://www.morphosource.org/Detail/MediaDetail/Show/media_id/24970 |  |
| *Xenopus laevis* (Daudin, 1802) | swimming | Emerson 1979 | CAS Herp 160540 | M25472-49896, http://www.morphosource.org/Detail/MediaDetail/Show/media_id/25472, doi:10.17602/M2/M49896 |  |

**TABLE A****3** Definition of landmarks and curves of semi-landmarks (fixed landmarks adopted from Engelkes et al., 2019).

| **Number** | **Type** | **Definition/remark** |
| --- | --- | --- |
| L1 | landmark | Cleithrum, dorsal point of anterior margin. |
| L2 | landmark | Cleithrum, ventral point of anterior margin. |
| L3 | landmark | Scapula, anterior point of dorsal margin. |
| L4 | landmark | Scapula, posterior point of dorsal margin. |
| L5 | landmark | Scapula, anterior point of posterior margin (extremal point of concave posterior margin). |
| L6 | landmark | Scapula, pars glenoidalis, point on dorsal margin of glenoid cavity that is closest to L12. |
| L7 | landmark | Scapula, pars glenoidalis, most posteroventral point of margin of glenoid cavity. |
| L8 | landmark | Scapula, pars glenoidalis, anteroventral point at the transition of ventral to anterior margin. |
| L9 | landmark | Scapula, dorsal point of medial notch between partes acromialis and glenoidalis. |
| L10 | landmark | Scapula, pars acromialis, ventral point of posterior margin of medial surface. |
| L11 | landmark | Scapula, ventral point of anterior margin. |
| L12 | landmark | Scapula, pars acromialis, ventral point of posterior margin of lateral surface. |
| L13 | landmark | Clavicula, lateralodorsal point of anterior margin. |
| L14 | landmark | Clavicula, anteromedial point of anterior margin. |
| L15 | landmark | Coracoid, lateral point of anterior margin. |
| L16 | landmark | Coracoid, medial point of anterior margin. |
| L17 | landmark | Coracoid, inflection point of anteroventral margin of glenoidal face. |
| L18 | landmark | Coracoid, lateral point of posterior margin. |
| L19 | landmark | Coracoid, lateral point of posterior margin. |
| C1 | curve | Cleithrum, anterior margin, between L1 and L2; 29 semi-landmarks plus endpoints. |
| C2 | curve | Scapula, laterodorsal margin, between L3 and L4; 21 semi-landmarks plus endpoints. |
| C3 | curve | Scapula, posterior margin, between L4 and L5; 29 semi-landmarks plus endpoints. |
| C4 | curve | Scapula, margin of glenoid cavity, between L6 and L7; 25 semi-landmarks plus endpoints. |
| C5 | curve | Scapula, anterior margin, between L3 and L11; 29 semi-landmarks plus endpoints. |
| C6 | curve | Clavicula, anterior margin, between L13 and L14; 29 semi-landmarks plus endpoints. |
| C7 | curve | Coracoid, anterior margin, between L15 and L16; 29 semi-landmarks plus endpoints. |
| C8 | curve | Coracoid, ventral margin of glenoidal face, between L17 and L18; 26 semi-landmarks plus endpoints. |
| C9 | curve | Coracoid, anterior margin, between L18 and L19; 29 semi-landmarks plus endpoints. |

# References cited in the Appendix

Barnes, W.J.P., Baum, M., Peisker, H. & Gorb, S.N. (2013) Comparative Cryo-SEM and AFM studies of hylid and rhacophorid tree frog toe pads. *Journal of Morphology*, **274** (12), 1384–1396.

Berneck, B.v.M., Segalla, M.V. & Haddad, C.F.B. (2017) A first observation of amplexus in *Aplastodiscus* (Anura; Hylidae). *Herpetology Notes*, **10**, 351–354.

Brown, L.E. & Crespo, E.G. (2000) Burrowing behavior of the midwife toads *Alytes cisternasii* and *Alytes obstetricans* (Anura, Discoglossidae). *Alytes*, **17** (3-4), 101–113.

Brown, L.E., Jackson, H.O. & Brown, J.R. (1972) Burrowing behavior of the chorus frog, *Pseudacris streckeri*. *Herpetologica*, **28** (4), 325–328.

Cevik, I.E., Baskale, E. & Kaya, U. (2008) A mark-recapture study of the fire-bellied toad (*Bombina bombina*) using photographic recognition and dye marking techniques in North-wester Turkey. *Acta Biologica Universitatis Daugavpiliensis*, **8** (1), 75–79.

Channing, A. (2001) *Amphibians of Central and Southern Africa*. Comstock Publishing Associates, Ithaca.

Cott, H.B. (1926) Observations on the life-habits of some batrachians and reptiles from the Lower Amazon: and a note on some mammals from Marajó Island. *Proceedings of the Zoological Society of London*, **96** (4), 1159–1178.

Emerson, S.B. (1976) Burrowing in frogs. *Journal of Morphology*, **149** (4), 437–458.

Emerson, S.B. (1979) The ilio-sacral articulation in frogs: form and function. *Biological Journal of the Linnean Scociety*, **11**, 153–168.

Emerson, S.B. & Koehl, M.A.R. (1990) The interaction of behavioral and morphological change in the evolution of a novel locomotor type: “flying” frogs. *Evolution*, **44** (8), 1931–1946.

Engelkes, K., Helfsgott, J., Hammel, J.U., Büsse, S., Kleinteich, T., Beerlink, A., Gorb, S.N. & Haas, A. (2019) Measurement error in μCT-based three-dimensional geometric morphometrics introduced by surface generation and landmark data acquisition. *Journal of Anatomy*, **235** (2), 357–378.

Enriquez-Urzelai, U., Montori, A., Llorente, G.A. & Kaliontzopoulou, A. (2015) Locomotor mode and the evolution of the hindlimb in Western Mediterranean anurans. *Evolutionary Biology*, **42** (2), 199–209.

Ferreira, R.C., Souza, A.A. de, Freitas, R.A., Campaner, M., Takata, C.S.A., Barrett, T.V., Shaw, J.J. & Teixeira, M.M.G. (2008) A phylogenetic lineage of closely related trypanosomes (Trypanosomatidae, Kinetoplastida) of anurans and sand flies (Psychodidae, Diptera) sharing the same ecotopes in brazilian amazonia. *The Journal of eukaryotic microbiology*, **55** (5), 427–435.

Haddad, C.F.B. & Sawaya, R.J. (2000) Reproductive modes of Atlantic forest hylid frogs: a general overview and the description of a new mode. *BIOTROPICA*, **32** (4), 862.

Hennigan, T. (2013) An initial estimate toward identifying and numbering the frog kinds on the Ark: order Anura. *Answers Research Journal*, **6**, 335–365.

Hertwig, I. & Sinsch, U. (1995) Comparative toe pad morphology in marsupial frogs (genus *Gastrotheca*): arboreal versus ground-dwelling species. *Copeia*, **1995** (1), 38.

Jorgensen, M.E. & Reilly, S.M. (2013) Phylogenetic patterns of skeletal morphometrics and pelvic traits in relation to locomotor mode in frogs. *Journal of Evolutionary Biology*, **26** (5), 929–943.

Laurent, R.F. (1964) Adaptive modifications in frogs of an isolated highland fauna in Central Africa. *Evolution*, **18** (3), 458.

Liem, D.S. (1973) A new genus of frog of the family Leptodactylidae from SE Queensland, Australia. *Memoirs of the Queensland Museum*, **16**, 459–470.

Littlejohn, M.J., Roberts, J.D., Watson, G.F. & Davies, M. (1993) Family Myobatrachidae. *Fauna of Australia* (eds C.G. Galsby, G.J.B. Ross & P.L. Beesley). Australian government Publishing Service, Melbourne.

Loveridge, J.P. & Withers, P.C. (1981) Metabolism and water balance of active and Cocooned African Bullfrogs *Pyxicephalus adspersus*. *Physiological Zoology*, **54** (2), 203–214.

Mai, C., Yu, J. & Liao, W. (2019) Ecological and Geographical Reasons for the Variation of Digestive Tract Length in Anurans. *Asian Herpetological Research*, **10** (4), 246-252.

Meserve, P.L. & Jaksic, F.M. (1991) Comparisons of terrestrial vertebrate assemblages in temperate rainforests of North and South America. *Revista Chilena de Historia Natural*, **64**, 511–535.

Myers, G.S. (1943) Rediscovery of the Philippine discoglossid frog, *Barbourula busuangensis*. *Copeia*, **1943** (3), 148.

Myers, CharleMyers, Charles W., Paolillo O., A., Daly, John W. s W. & Daly, J.W. (1991) Discovery of a malodorous and nocturnal frog in the family Dendrobatidae: phylogenetic significance of a new genus and species from the Venezuelan Andes. *American Museum Novitates*, **3002**, 1–20.

Natale, G.S., Alcalde, L., Herrera, R., Cajade, R., Schaefer, E.F., Marangoni, F. & Trudeau, V.L. (2011) Underwater acoustic communication in the macrophagic carnivorous larvae of *Ceratophrys ornata* (Anura: Ceratophryidae). *Acta Zoologica*, **92** (1), 46–53.

Nomura, F., Rossa-Feres, D.C. & Langeani, F. (2009) Burrowing behavior of *Dermatonotus muelleri* (Anura, Microhylidae) with reference to the origin of the burrowing behavior of Anura. *Journal of Ethology*, **27** (1), 195–201.

Pauly, G.B., Bernal, X. & Taylor, G.B. (2005) *Scinax ruber* (Red Snouted Treefrog). Arboreality and parachuting. *Herpetological Review*, **36** (3), 308–309.

Poynton, J.C. (1982) On species pairs among southern African amphibians. *African Zoology*, **17** (2), 67–74.

Roberts, W.E. (1994) Explosive breeding aggregations and parachuting in a neotropical frog, *Agalychnis saltator* (Hylidae). *Journal of Herpetology*, **28** (2), 193.

Savage, R.M. (1942) The burrowing and emergence of the Spade-Foot Toad, *Pelobates fuscus fuscus* Wagler. *Proceedings of the Zoological Society of London*, **A112** (1-2), 21–35.

Senevirathne, G., Thomas, A., Kerney, R., Hanken, J., Biju, S.D., Meegaskumbura, M. & Witten, P.E. (2016) From clinging to digging: the postembryonic skeletal ontogeny of the Indian Purple Frog, *Nasikabatrachus sahyadrensis* (Anura: Nasikabatrachidae). *PLoS ONE*, **11** (3), e0151114.

Shahrudin, S. (2017) Breeding activity of *Rhacophorus prominanus* Smith, 1924, from Peninsular Malaysia. *Herpetozoa*, **30** (1/2), 105–107.

Stewart, M.M. (1985) Arboreal habitat use and parachuting by a subtropical forest frog. *Journal of Herpetology*, **19** (3), 391–401.

Trueb, L. & Gans, C. (1983) Feeding specializations of the Mexican burrowing toad, *Rhinophrynus dorsalis* (Anura: Rhinophrynidae). *Journal of Zoology*, **199** (2), 189–208.

Worthy, T.H. (1987) Palaeoecological information concerning members of the frog genus *Leiopelma*: Leiopelmatidae in New Zealand. *Journal of the Royal Society of New Zealand*, **17** (4), 409–420.

Zug, G.R. (1978) Anuran locomotion-structure and function 2: jumping performance of semiaquatic, terrestrial and arboreal frogs. *Smithsonian Contributions to Zoology*, **276**, 1–31.
